# Discrimination and prediction of cultivation age and parts of *Panax ginseng* by Fourier-transform infrared spectroscopy combined with multivariate statistical analysis

**DOI:** 10.1371/journal.pone.0186664

**Published:** 2017-10-19

**Authors:** Byeong-Ju Lee, Hye-Youn Kim, Sa Rang Lim, Linfang Huang, Hyung-Kyoon Choi

**Affiliations:** 1 Bio-Integration Research Center for Nutra-Pharmaceutical Epigenetics, College of Pharmacy, Chung-Ang University, Seoul, Republic of Korea; 2 Institute of Medicinal Plant Development, Chinese Academy of Medical Sciences, Peking Union Medical College, Beijing, China; China University of Mining and Technology, CHINA

## Abstract

*Panax ginseng* C.A. Meyer is a herb used for medicinal purposes, and its discrimination according to cultivation age has been an important and practical issue. This study employed Fourier-transform infrared (FT-IR) spectroscopy with multivariate statistical analysis to obtain a prediction model for discriminating cultivation ages (5 and 6 years) and three different parts (rhizome, tap root, and lateral root) of *P*. *ginseng*. The optimal partial-least-squares regression (PLSR) models for discriminating ginseng samples were determined by selecting normalization methods, number of partial-least-squares (PLS) components, and variable influence on projection (VIP) cutoff values. The best prediction model for discriminating 5- and 6-year-old ginseng was developed using tap root, vector normalization applied after the second differentiation, one PLS component, and a VIP cutoff of 1.0 (based on the lowest root-mean-square error of prediction value). In addition, for discriminating among the three parts of *P*. *ginseng*, optimized PLSR models were established using data sets obtained from vector normalization, two PLS components, and VIP cutoff values of 1.5 (for 5-year-old ginseng) and 1.3 (for 6-year-old ginseng). To our knowledge, this is the first study to provide a novel strategy for rapidly discriminating the cultivation ages and parts of *P*. *ginseng* using FT-IR by selected normalization methods, number of PLS components, and VIP cutoff values.

## Introduction

*Panax ginseng* C.A. Meyer is one of the most valuable perennial herbs belonging to the family Araliaceae. *P*. *ginseng* has been used as a herbal remedy in eastern Asia for at least 2000 years due to its therapeutic effects [1], which are attributable to anticancer [2–4], antidiabetic [5,6], antistress [7,8], antioxidant [9,10], and immunomodulatory [11,12] activities. It was revealed that the pharmacological effects of *P*. *ginseng* vary according to its cultivation age and the parts used. Compared to young plants, aged *P*. *ginseng* plants exert stronger anticarcinogenic effects against lung tumors in mice [13]. The content of ginsenosides, which are the main active compounds of ginseng, was highest in the root hairs [14]. The different parts of ginseng including the tap root, lateral roots, rhizome head, and skin have different properties and medicinal values [15]. Quality assessments of *P*. *ginseng* root are important since its content of bioactive compounds varies with the cultivation age [16]. Authentication of *P*. *ginseng* has been mainly performed by assessing the ginsenoside content, morphological characteristics, smell, or taste [17]. Therefore, a more reliable objective method is needed for discriminating the cultivation ages and parts of *P*. *ginseng*.

Metabolomics can provide a comprehensive profile of all the metabolites present in an organism, and hence can be a valuable tool for quality control and discrimination [18,19]. Various studies have investigated the discrimination of *P*. *ginseng* by using liquid chromatography–quadrupole time-of-flight mass spectrometry [20], high-performance liquid chromatography [21,22], and nuclear magnetic resonance [23,24]. Fourier-transform infrared (FT-IR) spectroscopy is a rapid, reagentless, nondestructive, and high-throughput analytical technique that is widely used in metabolomics and metabolic fingerprinting [25]. Two-dimensional correlation infrared (2D-IR) and FT-IR spectroscopy have been used to discriminate plants with distinct geographical origins—from Beijing, Toronto, Vancouver, Wisconsin, and the American wild-type ginseng [26]. These two spectroscopy techniques were also used to discriminate various grades of cultivated ginseng species, namely transplanted, garden, and mountain cultivation [27]. Liu et al. successfully used FT-IR and 2D-IR spectroscopy to classify cultivated, mountain wild, and mountain cultivated ginseng based on their contents of starch, calcium oxalate, and fatty acids [28]. Yap et al. proposed discriminating Asian and American ginseng using an FT-IR-based protocol that utilized second-derivative spectral data between 2000 and 600 cm^–1^ [29]. Kwon et al. used FT-IR analysis of leaves of three cultivars to discriminate ginseng with different cultivation ages (1, 2, and 3 years) [30]. However, these previous studies that utilized FT-IR spectroscopy did not consider or optimize the data processing methods.

Prediction models constructed using multivariate statistical analysis are affected by various factors including the normalization method, the number of partial-least-squares (PLS) components, and the variable influence on projection (VIP) cutoff value. These factors can be adjusted to construct a more suitable model. Since FT-IR spectra can be affected by differences in sample thickness and particle size [31,32], the measured spectra should be normalized to reduce the variance and for standardization. The normalization methods are categorized as two types depending on the presence (minimum–maximum [min–max] normalization) or absence (area normalization and vector normalization) of reference peaks [33]. The prediction accuracy of a model is known to be affected by the number of PLS components, which means that the most appropriate number of PLS components needs to be determined in order to avoid the construction of underfitted (too few components) and overfitted (too many components) models [34]. In addition, VIP cutoff values can be selected to choose variables for optimizing PLS models [35].

To the best of our knowledge, no previous study has attempted to discriminate cultivation ages and parts of *P*. *ginseng* by using FT-IR spectroscopy based on optimal normalization methods, the number of PLS components, and VIP cutoff values. The objectives of this study were to propose optimal partial-least-squares regression (PLSR) models for discriminating ginseng samples according to cultivation ages and parts by selecting variables based on normalization methods, the number of PLS components, and VIP cutoff values.

## Materials and methods

### Plant materials and sample preparation

Twenty-four roots of *P*. *ginseng* C.A. Meyer (12 five-year-old and 12 six-year-old *P*. *ginseng* ‘Yunpoong’) were obtained from the Medicinal Crop Research Institute (Eumseong, Republic of Korea) in October 2014 (S1 Fig). The YP cultivar was registered in the Korea Seed and Variety Service (http://www.seed.go.kr) and cultivated in accordance with the “Ginseng GAP standard cultivation guide” developed by the Rural Development Administration (Republic of Korea).

The root samples of *P*. *ginseng* were washed with tap water, and were dissected into three parts based on ambient conditions: tap roots, rhizomes, and lateral roots. Each part from individual samples from each age group (5-year-old YP and 6-year-old YP) were instantly frozen in liquid nitrogen and stored at −80°C. After freeze-drying, the samples were ground into a fine powder by using mortar and pestle and stored at −80°C for further analysis.

### FT-IR analysis and spectral data preprocessing

*P*. *ginseng* powder (20 mg) was filtered through a sieve, and loaded onto IRTracer-100 spectrometer (Shimadzu Corp., Kyoto, Japan) equipped with an attenuated total reflection (ATR) accessory for recording the FT-IR spectrum. All of the FT-IR spectra were obtained using LabSolutions IR software (Shimadzu Corp., Kyoto, Japan). Sixty-four scans were recorded to improve signal-to-noise ratio and averaged for analytical results. Each spectrum was collected in wavenumber range from 4000 to 650 cm^-1^ with a spectral resolution of 4 cm^-1^. Six analytical replicates of FT-IR spectral data were obtained.

FT-IR spectra were differently processed using various normalization methods, such as area normalization, minnimum–maximum normalization, and vector normalization [33,36]. In vector normalization, all spectra were converted from transmittance to absorbance. FT-IR absorbance spectra was converted into first and second derivative (Savitzky-Golay derivative and 9 smoothing points) using OMNIC software (version 8.2.0.387; Thermo scientific, Waltham, Massachusetts, USA). In case of vector normalization, the Euclidean norm was used to normalize absorbance values of the spectra. Absorbance values of spectral data were divided by the Euclidean norm to calculate vector normalization value. In area and minimum-maximum normalizations, all spectra were converted from transmittance to absorbance, and then ATR correction was conducted using OMNIC software. The water vapor region (4000–3500 cm^-1^) and two CO_2_ region (CO_2_ region 1; 2442–2208 cm^-1^, CO_2_ region 2; 914–600 cm^-1^) were removed in all FT-IR spectral data using Microsoft Office Excel (version 2013; Microsoft, Redmond, WA, USA) [37]. For area normalization, each absorbance value at specific wavenumber was divided by total (integral) absorbance area of the spectrum. For min–max normalization, each absorbance value was divided by the difference between the highest and the lowest absorbance values.

### Multivariate statistical analysis

For the multivariate statistical analysis, the preprocessed FT-IR spectral data were imported into the SIMCA-P+ software (version 13.0; Umetrics, Umeå, Sweden) for principal component analysis (PCA), partial least squares-discriminant analysis (PLS-DA), and PLSR. All FT-IR spectral data were subjected to unit variance and pareto scaling. Cross-validation (internal validation) was used to minimize overfitting and give an estimation of the predictive capability of the PLS-DA models. The *Q*^*2*^ (predicted variation, “goodness of predictability”) and *R*^*2*^ (explained variation, “goodness of fit”) parameters were used to evaluate the models. Permutation test was performed 400 times using the SIMCA-P+ software. The PLSR models were validated to assess the predictive power with R^2^Y and Q^2^Y using cross-validation. Training set and test set were needed to perform cross-validation. Regression models were created by using training sets, and model’s predictive ability was verified by test sets. Grinded ginseng powder were used to obtain six replicated FT-IR spectral data. Five replicated data was used for PLS as a training set, and remained 1 data was employed as a test set for validation. After cross-validation, the statistical significance of PLSR models was assessed using permutation test parameters such as R^2^Y intercept and Q^2^Y intercept.

## Results and discussion

### Band assignment in FT-IR spectra

**Fig 1:**
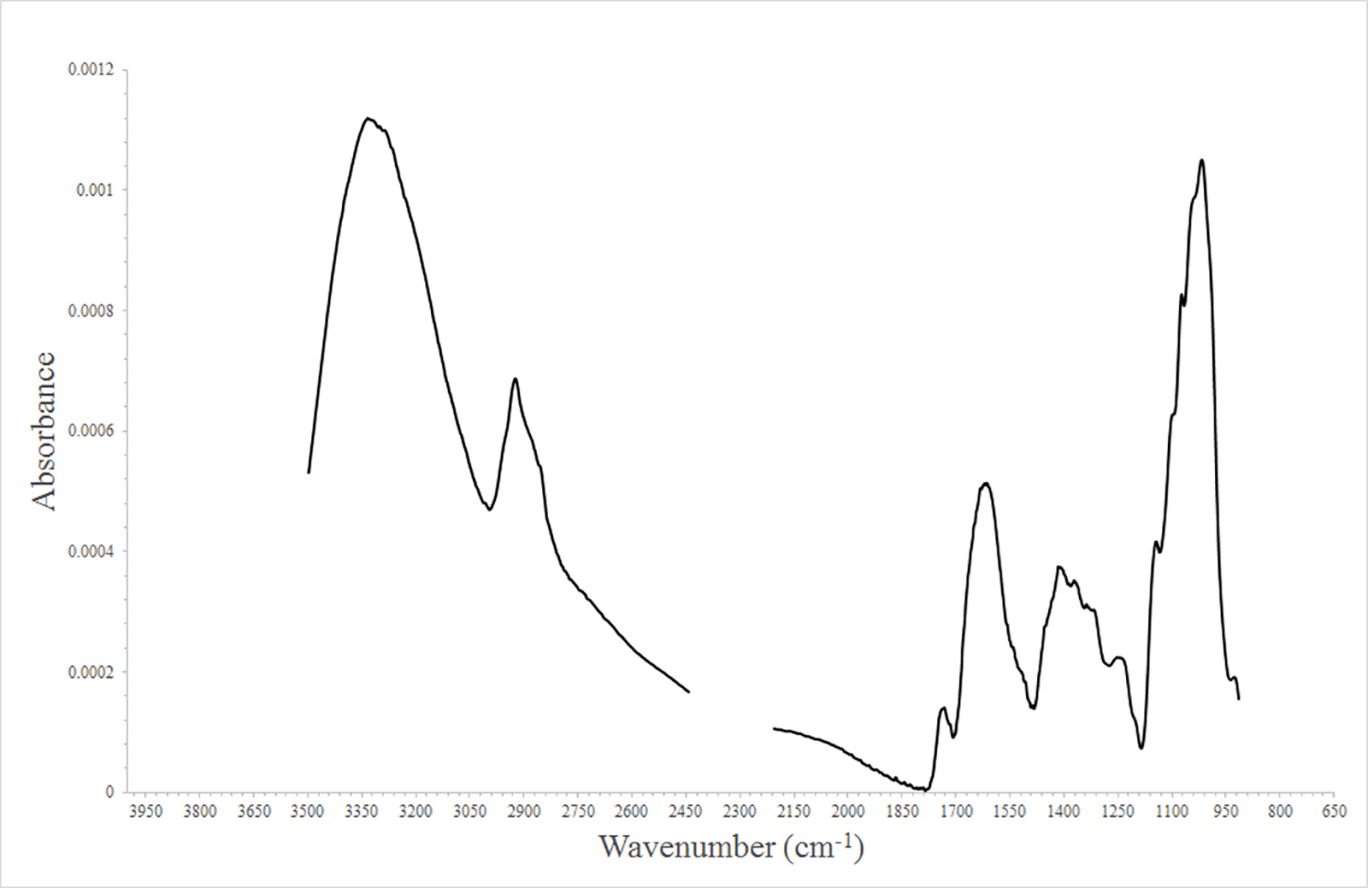
Representative FT-IR spectral data obtained after area normalization.

Various bands from representative FT-IR spectra of *P*. *ginseng* are shown in Fig 1, and Table 1 lists the assignment of each wave number to the corresponding functional groups. The band between 4000 and 3500 cm^–1^ was attributed to the stretching of O-H bonds in water vapor [37]. Proteins reportedly show nine types of amide bands in FT-IR spectra: amides A, B, and I–VII [38]. The 3335 cm^–1^ band was assigned to stretching of N-H bonds in proteins, which is known as the amide A band [39]. In addition, the 3335 cm^–1^ band can be assigned to the stretching of hydroxyl group in ginsenosides [40]. The 2923 cm^–1^ band was assigned to the stretching of C-H bonds in ginsenosides, fatty acids, lipids, and proteins [40,41]. The band between 2442 and 2208 cm^–1^ was due to the stretching of O-C-O bonds in carbon dioxide [37]. The band at 1733 cm^–1^ was due to stretching of C = O bonds of the carbonyl group [42]. The 1621 cm^–1^ band was assigned to calcium oxalate, which is abundant in *P*. *ginseng* roots [43,44]. The 1417 cm^–1^ band was attributable to the stretching of bonds in CH_3_ in lipids and aromatic compounds [39]. The band at 1373 cm^–1^ originated from the stretching of bonds in COO^−^and the bending of bonds in CH_3_ in lipids and proteins [45]. The band at 1253 cm^–1^ was assigned to amide III bands of proteins [46]. The strong band at 1018 cm^–1^ was attributed to the stretching of C-O-C bonds in polysaccharides [47]. The band between 914 and 600 cm^–1^ corresponded to the bending of O-C-O in carbon dioxide [37]. Water-vapor bands (4000 to 3500 cm^–1^) and CO_2_ bands (from 2442 to 2208 cm^–1^ and from 914 to 600 cm^–1^) were removed in order to avoid misleading results in the subsequent experiments. It can be assumed that ginseng root is mainly composed of saponin, polysaccharides, calcium oxalate, and lipids.

**Table 1:** Assignment of major bands in a representative Fourier-transform infrared (FT-IR) spectrum of *Panax ginseng* samples.

Wavenumber (cm^–1^)	Vibration	Suggested biomolecular assignment	Reference
4000–3500	O-H stretching	H_2_O	[37]
3335	O-H stretching	Hydroxyl group of ginsenosides	[40]
	N-H stretching	Amide A of proteins	[39]
2923	C-H stretching	C-H bond of ginsenosides	[40]
	C-H stretching (asymmetric)	CH_2_ in fatty acids, lipids, and proteins	[41]
		Methylene group of membrane phospholipids	[39]
2442–2208	O-C-O stretching	CO_2_	[37]
1733	C = O stretching	Carbonyl group and lipids	[42]
1621	OC = O stretching (asymmetric)	Calcium oxalate	[43]
	C-O and C-N stretching	Amide I of proteins	[41]
1417	CH_3_ stretching (asymmetric)	Lipids and aromatics	[39]
1373	COO^−^stretching (symmetric) and CH_3_ bending	Lipids and proteins	[45]
1253	N-H bending in plane and C-N stretching	Amide III of proteins	[46]
1018	C-O-C and CO stretching	Polysaccharides	[47]
	-C-O- stretching	Carbohydrates	[48]
914–600	O-C-O bending	CO_2_	[37]

### Determination of normalization, scaling methods, and number of PLS components

Permutation tests were performed to select normalization methods (area normalization, min–max normalization, and vector normalization), scaling methods (UV and Pareto), and the number of PLS components (from one to three PLS components) for discriminating the ages and parts of ginseng samples.

The permutation parameters for various normalization and scaling methods and numbers of PLS components of PLS-DA models for discriminating 5- and 6-year-old ginseng samples using tap root, rhizome, and lateral root are listed in S1, S2 and S3 Tables, respectively. The same parameters for discriminating the three parts of ginseng using 5- and 6-year-old samples are listed in S4 and S5 Tables, respectively.

Table 2 lists PLS-DA models selected from S1 to S5 Tables. R^2^Y and Q^2^Y indicate how well a model fitted the data and how well it predicted the results of other experiments, respectively. Both the R^2^Y and Q^2^Y values range between 0 and 1.0. A higher R^2^Y value in a PLS-DA model indicates a better model fit. Q^2^Y values within the range of 0.5–0.9 are considered to indicate good predictability, while those of 0.9–1.0 indicate excellent predictability. The R^2^Y and Q^2^Y intercepts are obtained in a permutation test; in valid models these parameters must be less than 0.4 and 0.05, respectively [49]. Among valid PLS-DA models satisfying R^2^Y and Q^2^Y intercept values, those models obtained by area or min–max normalization and using two PLS components showed higher R^2^Y and Q^2^Y values for discriminating 5- and 6-year-old ginseng samples using tap root, rhizome, and lateral root. When vector normalization was employed to construct the PLS-DA model, the use of one PLS component produced higher R^2^Y and Q^2^Y values.

**Table 2:** Selection of partial-least-squares–discriminant analysis (PLS-DA) models according to various normalization and scaling methods and numbers of PLS components for discriminating cultivation ages and parts of *P*. *ginseng* samples.

Normalization method	Scaling	R^2^Y	Q^2^Y	R^2^Y intercept	Q^2^Y Intercept	Number of components
**5- vs. 6-year-old TR**						
Area	UV	0.904	0.719	0.343	–0.373	2
Min–max	UV	0.870	0.832	0.265	–0.389	2
Vector (first)	UV	0.961	0.855	0.390	–0.275	1
Vector (second)	Par	0.973	0.907	0.119	–0.325	1
**5- vs. 6-year-old RH**						
Area	UV	0.880	0.816	0.384	–0.290	2
Min–max	Par	0.841	0.722	0.313	–0.231	2
Vector (first)	Par	0.725	0.478	0.360	–0.141	1
Vector (second)	Par	0.887	0.586	0.209	–0.164	1
**5- vs. 6-year-old LR**						
Area	Par	0.923	0.798	0.391	–0.280	2
Min–max	Par	0.939	0.723	0.391	–0.209	2
Vector (first)	Par	0.774	0.672	0.285	–0.233	1
Vector (second)	Par	0.677	0.417	0.533	–0.109	1
**5-year-old TR vs. RH vs. LR**						
Area	Par	0.866	0.771	0.270	–0.312	3
Min–max	Par	0.826	0.544	0.264	–0.243	3
Vector (first)	Par	0.908	0.754	0.328	–0.370	3
Vector (second)	Par	0.915	0.862	0.363	–0.349	3
**6-year-old TR vs. RH vs. LR**						
Area	Par	0.889	0.758	0.256	–0.370	3
Min–max	Par	0.849	0.681	0.288	–0.327	3
Vector (first)	UV	0.889	0.800	0.362	–0.340	2
Vector (second)	Par	0.678	0.501	0.265	–0.317	2

TR, tap root; RH, rhizome; LR, lateral root; Min–max, minimum–maximum; Vector (first), vector normalization applied after the first differentiation; Vector (second), vector normalization applied after the second differentiation; UV, unit variance; Par, Pareto.

To discriminate the three parts (tap root, rhizome, and lateral root) of 5-year-old ginseng samples, higher R^2^Y and Q^2^Y values were obtained by using any of the normalization methods when three PLS components were used to establish the models. To discriminate the three parts of 6-year-old ginseng samples, higher R^2^Y and Q^2^Y values were obtained by using three PLS components with area or min–max normalization, whereas models constructed with two PLS components showed higher R^2^Y and Q^2^Y values by vector normalization.

### Development of a PLSR model for predicting the cultivation ages of ginseng

We constructed PLSR models to predict the ages and parts of ginseng samples based on the selected normalization method and the number of PLS components. In addition, various VIP cutoff values were used to select variables for constructing the prediction models. PLSR models were constructed based on data from the training set, and the constructed models were evaluated using the test set (which was independent from training set). Root-mean-square error of estimation (RMSEE) values were obtained from PLSR models constructed based on training sets. These values were then evaluated to determine the accuracy of PLSR models. Root-mean-square error of prediction (RMSEP) values were used to assess the predictability of the models. The values of RMSEE and RMSEP range between 0 and 1, with smaller values indicating higher accuracy and predictability of the models.

As listed in S6–S13 Tables, various VIP cutoff values were tested in order to construct better prediction models based on the RMSEP values among those satisfying the R^2^Y and Q^2^Y intercept values. S6–S9 Tables list the prediction models for discriminating between 5- and 6-year-old ginseng samples. The best models for each part of the *P*. *ginseng* samples among S6–S9 Tables are listed in Table 3. For tap root, the PLS-DA model constructed by vector normalization applied after the second differentiation with a VIP cutoff of 1.0 showed the lowest RMSEP value of 0.044 (0.528 months) along with a higher R^2^Y value of 0.981 and a Q^2^Y value of 0.970 (S2 Fig). For rhizome, min–max normalization with a VIP cutoff of 1.3 was employed to construct the best PLSR model, which showed the lowest RMSEP value of 0.036 (0.432 months) when discriminating between the 5- and 6-year-old ginseng samples (S3 Fig). For lateral root, the PLSR model using area normalization with a VIP cutoff of 1.3 showed a RMSEP value of 0.096 (1.152 months), which was higher than those for tap root and rhizome (S4 Fig).

**Table 3:** Selected normalization and variable influence on projection (VIP) cutoff values for model construction for discriminating 5- and 6-year-old ginseng samples and permutation parameters derived from the partial-least-squares regression (PLSR) prediction models.

Normalization method	VIP cutoff	Total wavenumbers	RMSEE (months)	RMSEP (months)	R^2^Y	Q^2^Y	R^2^Y intercept	Q^2^Y intercept	Number of components
**5- vs. 6-year-old TR (UV scaling)**									
**Vector (second)**	1.0	552	0.077 (0.924)	0.044 (0.528)	0.981	0.970	–0.064	–0.369	1
**5- vs. 6-year-old RH (UV scaling)**									
**Min–max**	1.3	112	0.198 (2.376)	0.036 (0.432)	0.890	0.788	0.201	–0.389	2
**5- vs. 6-year-old LR (UV scaling)**									
**Area**	1.3	262	0.171 (2.052)	0.096 (1.152)	0.918	0.806	0.231	–0.296	2

TR, tap root; RH, rhizome; LR, lateral root; RMSEE, root-mean-square error of estimation; RMSEP, root-mean-square error of prediction; UV, unit variance.

Table 3 indicates that two prediction models using tap root and rhizome were suitable for discriminating 5- and 6-year-old ginseng samples. However, the RMSEE, R^2^Y, and Q^2^Y values of PLSR models when using tap root were better than for those when using rhizome. Thus, the PLSR model using tap root can be considered as the most suitable model for discriminating the cultivation age. However, the rhizome is generally removed before using *P*. *ginseng* root due to its emetic effects [50]. The rhizome has economically lower worth than tap root because of this adverse effect. The rhizome of *P*. *ginseng* samples could be an alternative resource to the tap root for discriminating 5- and 6-year-old ginseng samples without the concern of economical loss.

### Development of a PLSR model for predicting the parts of ginseng

S10–S13 Tables list various prediction models for discriminating ginseng parts, among which **Table 4** lists the best models for discriminating 5- and 6-year-old ginseng parts. For predicting the various parts of 5-year-old ginseng samples, the PLS-DA model constructed by vector normalization after the first differentiation and with a VIP cutoff of 1.5 and two PLS components showed the lowest RMSEP value of 0.161 along with a higher R^2^Y value of 0.950 and a Q^2^Y value of 0.913 (S5 Fig). These values suggest that the model had excellent predictive abilities.

**Table 4:** Selected normalization and variable influence on projection (VIP) cutoff values for model construction for discriminating various parts of ginseng samples, and the permutation parameters derived from the PLSR prediction models.

Normalization method	VIP cutoff	Total wavenumbers	RMSEE	RMSEP	R^2^Y	Q^2^Y	R^2^Y intercept	Q^2^Y intercept	Number of components
**5-year-old TR vs. RH vs. LR (UV scaling)**									
**Vector (first)**	1.5	23	0.204	0.161	0.950	0.913	0.352	–0.223	2
**6-year-old TR vs. RH vs. LR (Par scaling)**									
**Vector (second)**	1.3	258	0.337	0.185	0.864	0.764	0.363	–0.321	2

TR, tap root; RH, rhizome; LR, lateral root; RMSEE, root-mean-square error of estimation; RMSEP, root-mean-square error of prediction; UV, unit variance; Par, Pareto.

For discriminating various parts of 6-year-old ginseng samples, vector normalization applied after the second differentiation and with a VIP cutoff of 1.3 and two PLS components was the best model. This model showed the lowest RMSEP value of 0.185 and a higher R^2^Y value of 0.864 and a Q^2^Y value of 0.764 (S6 Fig). It is generally difficult to determine the parts of ginseng that have been used to produce powdered ginseng products. The content of ginsenosides, which are the main compound in ginseng, is higher in lateral roots than in the tap root [51]. Even if commercial ginseng products comprise only 6-year-old ginseng, the efficacy and composition of ginseng samples might differ with the ginseng parts. Therefore, the PLSR model for discriminating the various parts of ginseng could be useful from both academic and commercial points of view.

## Conclusions

**Fig 2:**
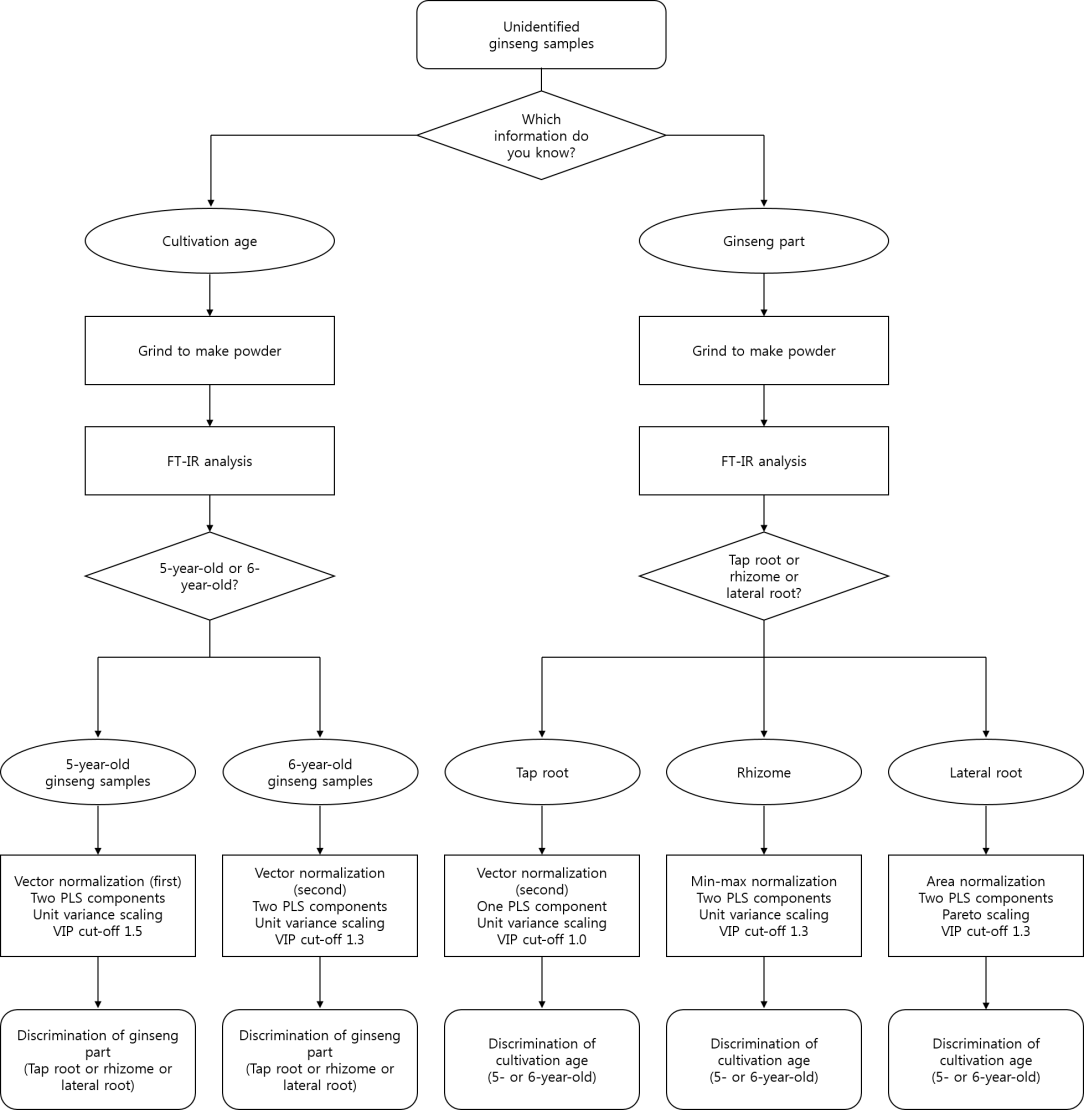
Flow chart to discriminate cultivation ages and parts of ginseng. VIP, variable influence on projection.

This study employed FT-IR analysis combined with multivariate statistical analysis to discriminate 5- and 6-year-old ginseng samples as well as three parts of ginseng plants. The focus was on 5- and 6-year-old ginseng roots since they constitute most of the commercially available ginseng products. For discriminating cultivation age and different parts, various conditions were selected including the number of PLS components, normalization methods, and VIP cutoff value, as shown in Fig 2. The best prediction model for discriminating 5- and 6-year-old ginseng was obtained using the tap root. Vector normalization applied after the second differentiation, one PLS component, and a VIP cutoff of 1.0 were suggested to be optimal (based on the lowest RMSEP value) for the construction of this prediction model. In addition, for discriminating the three parts of *P*. *ginseng*, the optimized PLSR models were established by vector normalization, two PLS components, and selecting variables based on VIP cutoff values of 1.5 (for 5-year-old ginseng) and 1.3 (for 6-year-old ginseng).

To our knowledge, this is the first study to determine suitable normalization methods and the number of PLS components of FT-IR spectral data in the development of PLSR models to discriminate 5- and 6-year-old ginseng samples and various ginseng parts. The information obtained in this study provides a solid foundation for further studies using various cultivars, cultivation methods, and geographic origins of ginseng samples to construct commercially applicable discrimination and prediction models.

## Supporting information

S1 Fig External appearance characteristics of *Panax ginseng* ‘Yunpoong’ sample with different parts used in this study. *Panax ginseng* is composed of three parts.
(TIF)

S2 Fig **Score plot derived from PLSR model of *Panax ginseng* tap root (TR) based on variables with VIP values over 1.0 (A), permutation testing plot (B), and correlation plot using training set (C) and test set (D).** Second differentiation, vector normalization, and unit variance scaling were used in FT-IR spectrum. PLSR, partial least squares regression; VIP, variable influence on projection; RMSEE, root mean squared error of estimation; RMSEP, root mean squared error of prediction.
(TIF)

S3 Fig **Score plot derived from PLSR model of *Panax ginseng* rhizome (RH) based on variables with VIP values over 1.3 (A), permutation testing plot (B), and correlation plot using training set (C) and test set (D).** Minimum-maximum normalization and unit variance scaling were used in FT-IR spectrum. PLSR, partial least squares regression; VIP, variable influence on projection; RMSEE, root mean squared error of estimation; RMSEP, root mean squared error of prediction.
(TIF)

S4 Fig **Score plot derived from PLSR model of *Panax ginseng* lateral root (LR) based on variables with VIP values over 1.3 (A), permutation testing plot (B) and correlation plot using training set (C), test set (D).** Area normalization and unit variance scaling were used in FT-IR spectrum. PLSR, partial least squares regression; VIP, variable influence on projection; RMSEE, root mean squared error of estimation; RMSEP, root mean squared error of prediction.
(TIF)

S5 Fig **Score plot derived from PLSR model of 5-year-old *Panax ginseng* based on variables with VIP values over 1.5 (A), permutation testing plot (B), and correlation plot using training set (C) and test set (D).** First differentiation, vector normalization, and unit variance scaling were used in FT-IR spectrum. PLSR, partial least squares regression; VIP, variable influence on projection; RMSEE, root mean squared error of estimation; RMSEP, root mean squared error of prediction.
(TIF)

S6 Fig **Score plot derived from PLSR model of 6-year-old *Panax ginseng* based on variables with VIP values over 1.3 (A), permutation testing plot (B), and correlation plot using training set (C) and test set (D).** Second differentiation, vector normalization, and pareto scaling were used in FT-IR spectrum. PLSR, partial least squares regression; VIP, variable influence on projection; RMSEE, root mean squared error of estimation; RMSEP, root mean squared error of prediction.
(TIF)

S1 Table PLS-DA model parameters according to the number of components (one to three components), normalization (area, minimum–maximum, and vector normalization) and scaling methods (unit variance and pareto) for differentiation of cultivation ages of *Panax ginseng* using tap root (TR). For vector normalization, first and second differentiations were applied. PLS-DA, partial least squares discriminant analysis; Min-max, minimum-maximum; UV, unit variance; Par, pareto.
(DOCX)

S2 Table PLS-DA model parameters according to the number of components (one to three components), normalization (area, minimum–maximum, and vector normalization), and scaling methods (unit variance and pareto) for differentiation of cultivation ages of *Panax ginseng* using rhizome (RH). For vector normalization, first and second differentiations were applied. PLS-DA, partial least squares discriminant analysis; Min-max, minimum-maximum; UV, unit variance; Par, pareto.
(DOCX)

S3 Table PLS-DA model parameters according to the number of components (one to three components), normalization (area, minimum–maximum, and vector normalization), and scaling methods (unit variance and pareto) for differentiation of cultivation ages of *Panax ginseng* using lateral root (LR). For vector normalization, first and second differentiations were applied. PLS-DA, partial least squares discriminant analysis; Min-max, minimum-maximum; UV, unit variance; Par, pareto.
(DOCX)

S4 Table PLS-DA model parameters according to the number of components (one to three components), normalization (area, minimum–maximum, and vector normalization), and scaling methods (unit variance and pareto) for differentiation of ginseng parts using 5-year-old *Panax ginseng*. For vector normalization, first and second differentiations were applied. PLS-DA, partial least squares discriminant analysis; Min-max, minimum-maximum; UV, unit variance; Par, pareto.
(DOCX)

S5 Table PLS-DA model parameters according to the number of components (one to three components), normalization (area, minimum-maximum, and vector normalization), and scaling methods (unit variance and pareto) for differentiation of ginseng parts using 6-year-old *Panax ginseng*. For vector normalization, first and second differentiations were applied. PLS-DA, partial least squares discriminant analysis; Min-max, minimum-maximum; UV, unit variance; Par, pareto.
(DOCX)

S6 Table List of permutation parameters obtained by variables selected by various variable influence on projection (VIP) cutoff values and scaling methods. Area normalization and two PLS components were used for discriminating between 5- and 6-year-old ginseng samples. TR, tap root; RH, rhizome; LR, lateral root; RMSEE, root mean squared error of estimation; RMSEP, root mean squared error of prediction; UV, unit variance; Par, pareto.
(DOCX)

S7 Table List of permutation parameters obtained by variables selected by various variable influence on projection (VIP) cutoff values and scaling methods. Minimum-maximum normalization and two PLS components were used for discriminating between 5- and 6-year-old ginseng samples. TR, tap root; RH, rhizome; LR, lateral root; RMSEE, root mean squared error of estimation; RMSEP, root mean squared error of prediction; UV, unit variance; Par, pareto.
(DOCX)

S8 Table List of permutation parameters obtained by variables selected by various variable influence on projection (VIP) cutoff values and scaling methods. Vector normalization after first differentiation and one PLS component were used for discriminating between 5- and 6-year-old ginseng samples. TR, tap root; RH, rhizome; LR, lateral root; RMSEE, root mean squared error of estimation; RMSEP, root mean squared error of prediction; UV, unit variance; Par, pareto.
(DOCX)

S9 Table List of permutation parameters obtained by variables selected by various variable influence on projection (VIP) cutoff values and scaling methods. Vector normalization after second differentiation and one PLS component were used for discriminating between 5- and 6-year-old ginseng samples. TR, tap root; RH, rhizome; LR, lateral root; RMSEE, root mean squared error of estimation; RMSEP, root mean squared error of prediction; UV, unit variance; Par, pareto.
(DOCX)

S10 Table List of permutation parameters obtained by variables selected by various variable influence on projection (VIP) cutoff values and scaling methods. Area normalization and three PLS components were used for discriminating ginseng samples from three parts (tap root, rhizome, lateral root). TR, tap root; RH, rhizome; LR, lateral root; RMSEE, root mean squared error of estimation; RMSEP, root mean squared error of prediction; UV, unit variance; Par, pareto.
(DOCX)

S11 Table List of permutation parameters obtained by variables selected by various variable influence on projection (VIP) cutoff values and scaling methods. Minimum–maximum normalization and three PLS components were used for discriminating ginseng samples from three parts (tap root, rhizome, lateral root). TR, tap root; RH, rhizome; LR, lateral root; RMSEE, root mean squared error of estimation; RMSEP, root mean squared error of prediction; UV, unit variance; Par, pareto.
(DOCX)

S12 Table List of permutation parameters obtained by variables selected by various variable influence on projection (VIP) cutoff values and scaling methods. Vector normalization after first differentiation and two PLS components were used for discriminating ginseng samples from three parts (tap root, rhizome, lateral root). TR, tap root; RH, rhizome; LR, lateral root; RMSEE, root mean squared error of estimation; RMSEP, root mean squared error of prediction; UV, unit variance; Par, pareto.
(DOCX)

S13 Table List of permutation parameters obtained by variables selected by various variable influence on projection (VIP) cutoff values and scaling methods. Vector normalization after second differentiation and two PLS components were used for discriminating ginseng samples from three parts (tap root, rhizome, lateral root). TR, tap root; RH, rhizome; LR, lateral root; RMSEE, root mean squared error of estimation; RMSEP, root mean squared error of prediction; UV, unit variance; Par, pareto.
(DOCX)
