# Supplementary figures and images for: Discrimination and prediction of cultivation age and parts of Panax ginseng by Fourier-transform infrared spectroscopy combined with multivariate statistical analysis
Source: PLoS One. 2017 Oct 19;12(10):e0186664. doi: 10.1371/journal.pone.0186664 (PMC5648215; doi:10.1371/journal.pone.0186664)

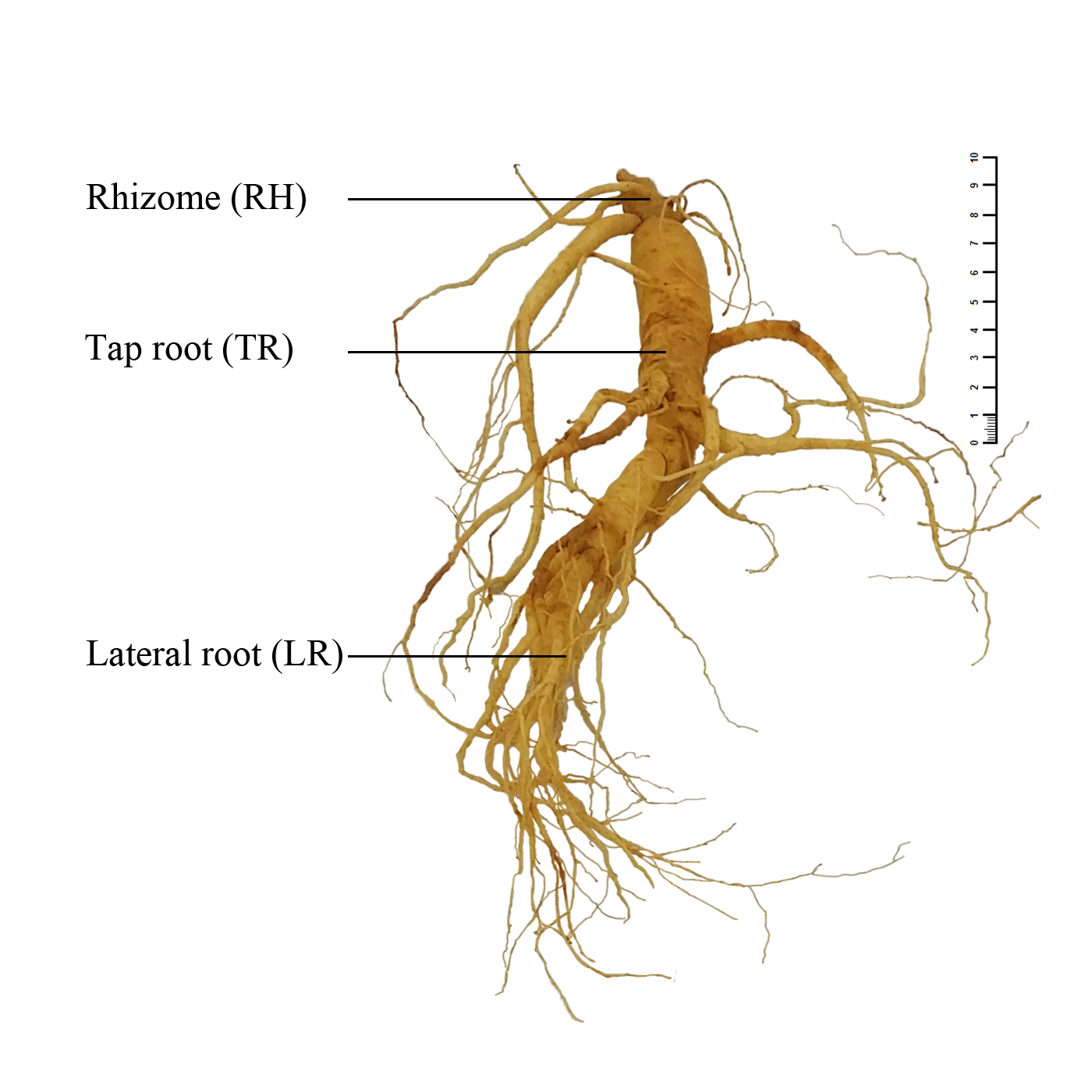

Supplement: S1 Fig — Panax ginseng is composed of three parts. (TIF) [file pone.0186664.s001.tif]

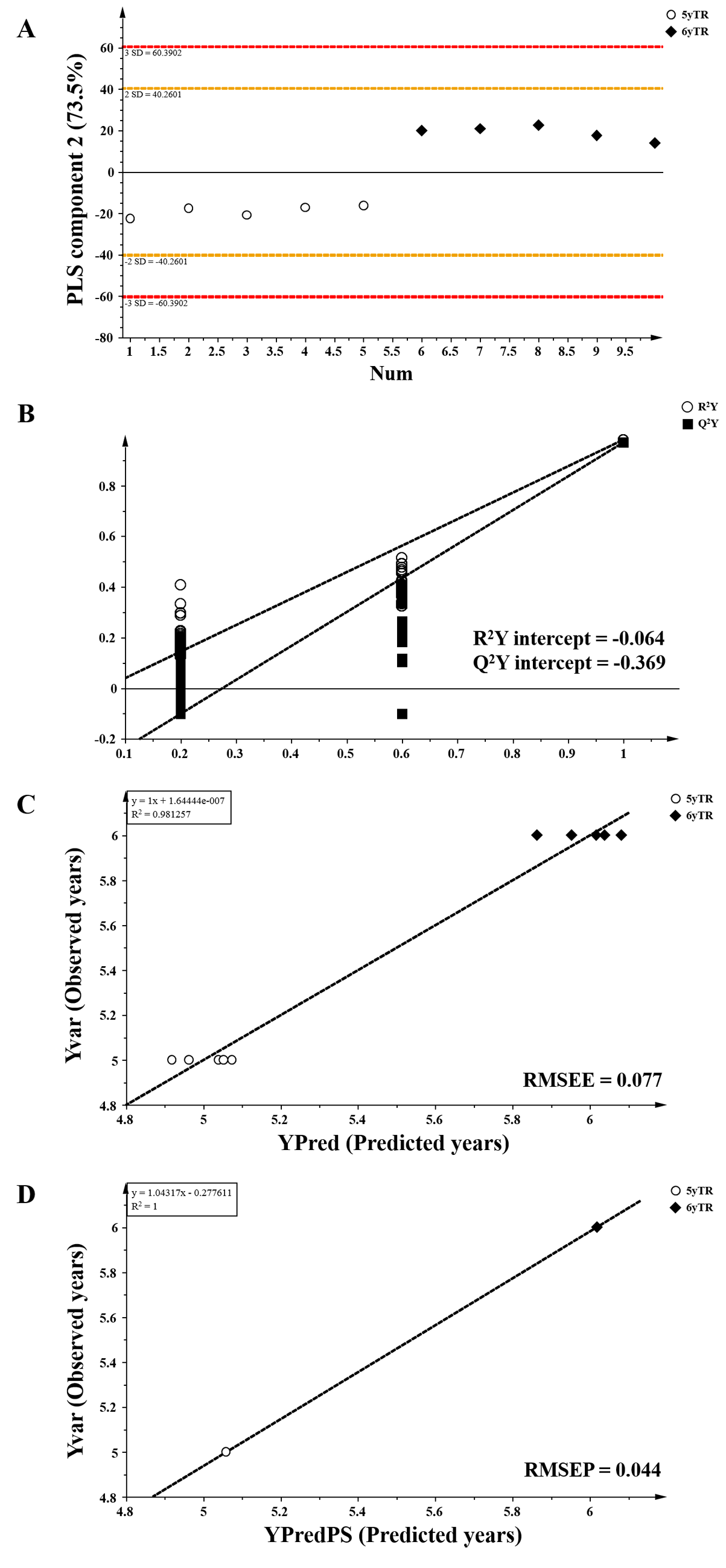

Supplement: S2 Fig — Score plot derived from PLSR model of Panax ginseng tap root (TR) based on variables with VIP values over 1.0 (A), permutation testing plot (B), and correlation plot using training set (C) and test set (D). Second differentiation, vector normalization, and unit variance scaling were used in FT-IR spectrum. PLSR, partial least squares regression; VIP, variable influence on projection; RMSEE, root mean squared error of estimation; RMSEP, root mean squared error of prediction. (TIF) [file pone.0186664.s002.tif]

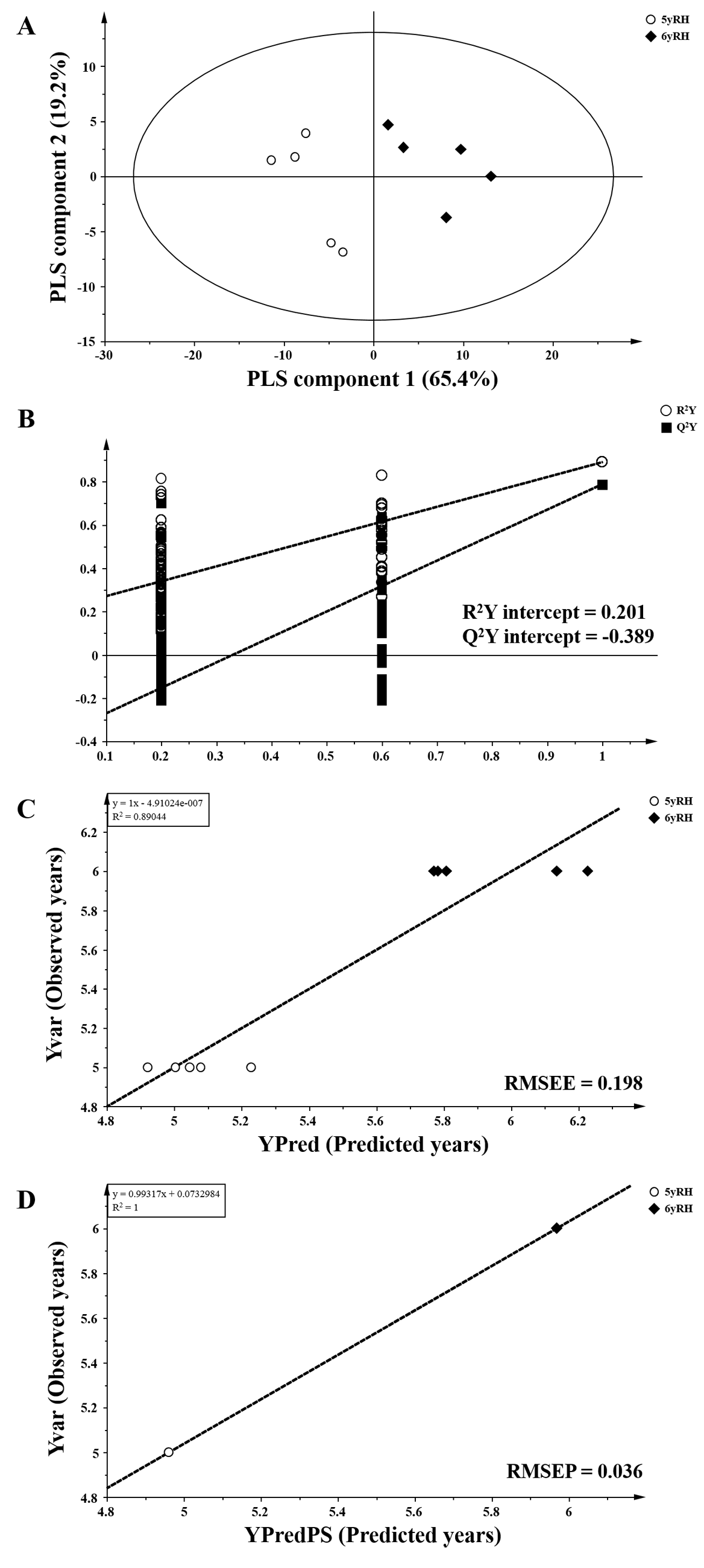

Supplement: S3 Fig — Score plot derived from PLSR model of Panax ginseng rhizome (RH) based on variables with VIP values over 1.3 (A), permutation testing plot (B), and correlation plot using training set (C) and test set (D). Minimum-maximum normalization and unit variance scaling were used in FT-IR spectrum. PLSR, partial least squares regression; VIP, variable influence on projection; RMSEE, root mean squared error of estimation; RMSEP, root mean squared error of prediction. (TIF) [file pone.0186664.s003.tif]

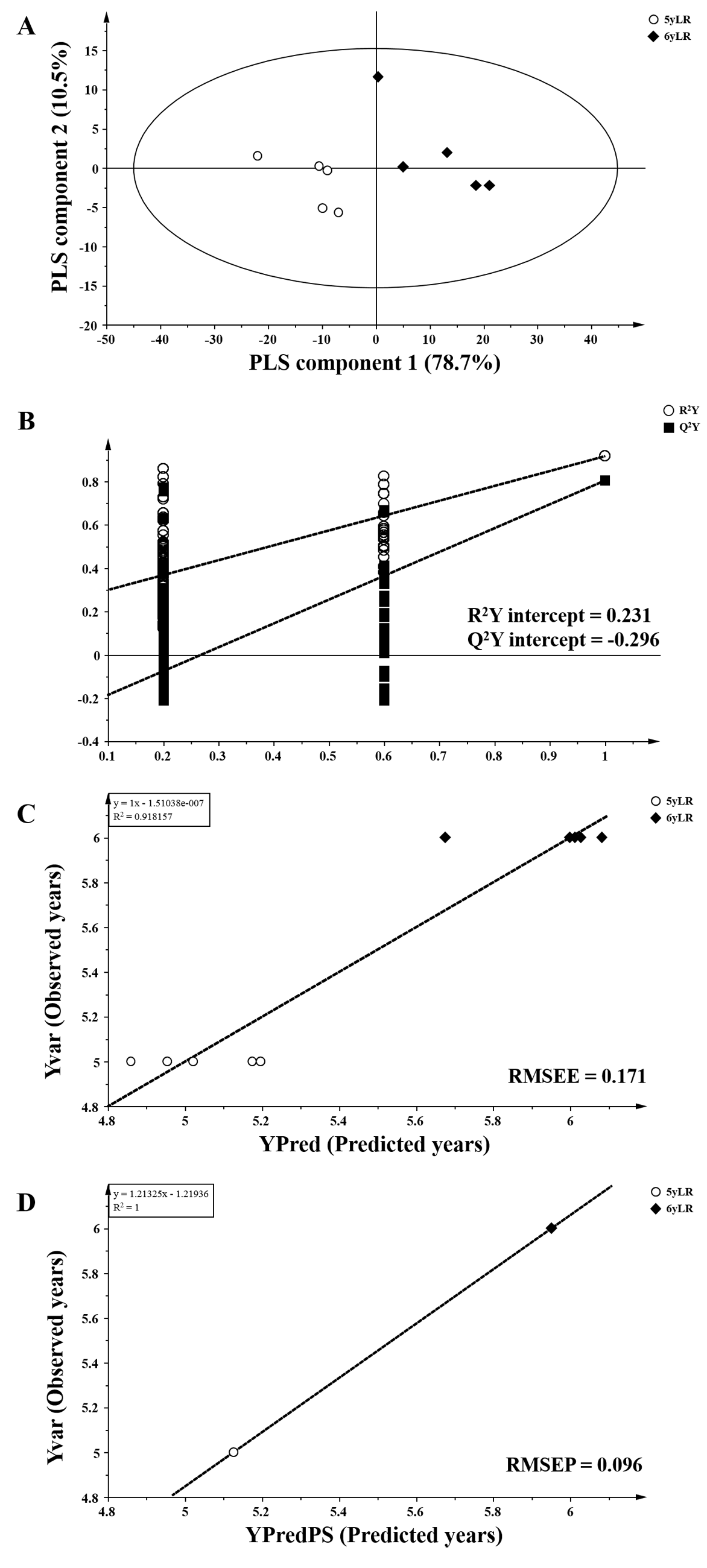

Supplement: S4 Fig — Score plot derived from PLSR model of Panax ginseng lateral root (LR) based on variables with VIP values over 1.3 (A), permutation testing plot (B) and correlation plot using training set (C), test set (D). Area normalization and unit variance scaling were used in FT-IR spectrum. PLSR, partial least squares regression; VIP, variable influence on projection; RMSEE, root mean squared error of estimation; RMSEP, root mean squared error of prediction. (TIF) [file pone.0186664.s004.tif]

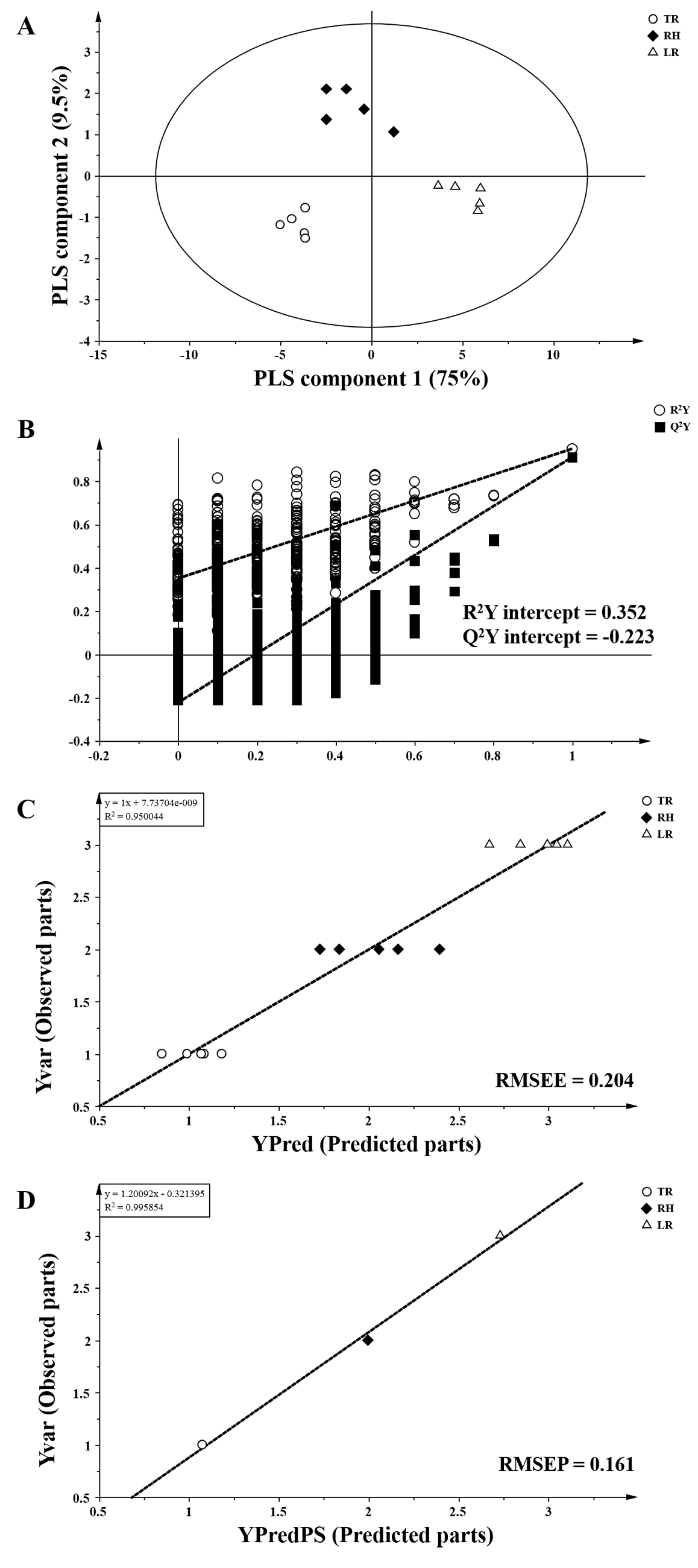

Supplement: S5 Fig — Score plot derived from PLSR model of 5-year-old Panax ginseng based on variables with VIP values over 1.5 (A), permutation testing plot (B), and correlation plot using training set (C) and test set (D). First differentiation, vector normalization, and unit variance scaling were used in FT-IR spectrum. PLSR, partial least squares regression; VIP, variable influence on projection; RMSEE, root mean squared error of estimation; RMSEP, root mean squared error of prediction. (TIF) [file pone.0186664.s005.tif]

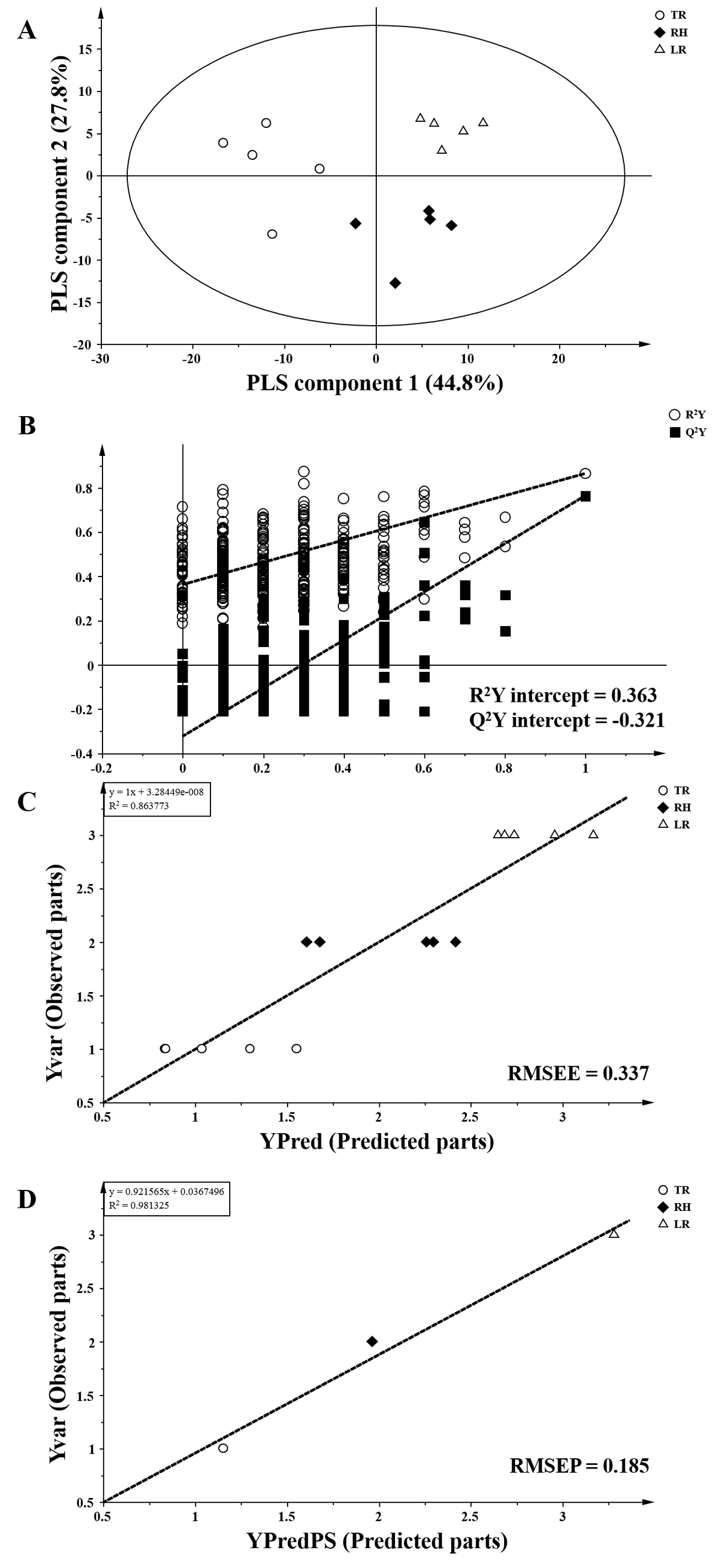

Supplement: S6 Fig — Score plot derived from PLSR model of 6-year-old Panax ginseng based on variables with VIP values over 1.3 (A), permutation testing plot (B), and correlation plot using training set (C) and test set (D). Second differentiation, vector normalization, and pareto scaling were used in FT-IR spectrum. PLSR, partial least squares regression; VIP, variable influence on projection; RMSEE, root mean squared error of estimation; RMSEP, root mean squared error of prediction. (TIF) [file pone.0186664.s006.tif]
