# Supplementary material for: Discrimination and prediction of cultivation age and parts of Panax ginseng by Fourier-transform infrared spectroscopy combined with multivariate statistical analysis
Source: PLoS One. 2017 Oct 19;12(10):e0186664. doi: 10.1371/journal.pone.0186664 (PMC5648215; doi:10.1371/journal.pone.0186664)
Supplement: S1 Table — For vector normalization, first and second differentiations were applied. PLS-DA, partial least squares discriminant analysis; Min-max, minimum-maximum; UV, unit variance; Par, pareto. (DOCX) [file pone.0186664.s007.docx]

**S1 Table. PLS-DA model parameters according to the number of components (one to three components), normalization (area, minimum–maximum, and vector normalization) and scaling methods (unit variance and pareto) for differentiation of cultivation ages of *Panax ginseng* using tap root (TR).**

|  | **Normalization method** | **Scaling** | **R^2^Y** | **Q^2^Y** | **R^2^Y intercept** | **Q^2^Y intercept** |
| --- | --- | --- | --- | --- | --- | --- |
| **One component** | | | | | | |
| 5- vs. 6-year-old TR | Area | UV | 0.868 | 0.745 | 0.171 | -0.231 |
|  |  | Par | 0.846 | 0.681 | 0.134 | -0.230 |
|  | Min-max | UV | 0.724 | 0.710 | 0.157 | -0.217 |
|  |  | Par | 0.520 | 0.459 | 0.170 | -0.131 |
|  | Vector (first) | UV | 0.961 | 0.855 | 0.390 | -0.275 |
|  |  | Par | 0.945 | 0.873 | 0.202 | -0.273 |
|  | Vector (second) | UV | 0.986 | 0.918 | 0.415 | -0.306 |
|  |  | Par | 0.973 | 0.907 | 0.119 | -0.325 |
| **Two components** | | | | | | |
| 5- vs. 6-year-old TR | Area | UV | 0.904 | 0.719 | 0.343 | -0.373 |
|  |  | Par | 0.916 | 0.649 | 0.332 | -0.346 |
|  | Min-max | UV | 0.870 | 0.832 | 0.265 | -0.389 |
|  |  | Par | 0.842 | 0.728 | 0.274 | -0.305 |
|  | Vector (first) | UV | 0.993 | 0.932 | 0.695 | -0.366 |
|  |  | Par | 0.976 | 0.885 | 0.507 | -0.379 |
|  | Vector (second) | UV | 0.996 | 0.933 | 0.870 | 0.155 |
|  |  | Par | 0.986 | 0.897 | 0.611 | -0.311 |
| **Three components** | | | | | | |
| 5- vs. 6-year-old TR | Area | UV | 0.922 | 0.691 | 0.526 | -0.350 |
|  |  | Par | 0.933 | 0.614 | 0.464 | -0.384 |
|  | Min-max | UV | 0.908 | 0.837 | 0.405 | -0.493 |
|  |  | Par | 0.870 | 0.701 | 0.449 | -0.413 |
|  | Vector (first) | UV | 0.999 | 0.978 | 0.832 | -0.100 |
|  |  | Par | 0.991 | 0.932 | 0.657 | -0.457 |
|  | Vector (second) | UV | 1.000 | 0.982 | 0.986 | 0.732 |
|  |  | Par | 0.998 | 0.972 | 0.898 | 0.420 |

For vector normalization, first and second differentiations were applied. PLS-DA, partial least squares discriminant analysis; Min-max, minimum-maximum; UV, unit variance; Par, pareto.
