# Supplementary material for: Discrimination and prediction of cultivation age and parts of Panax ginseng by Fourier-transform infrared spectroscopy combined with multivariate statistical analysis
Source: PLoS One. 2017 Oct 19;12(10):e0186664. doi: 10.1371/journal.pone.0186664 (PMC5648215; doi:10.1371/journal.pone.0186664)
Supplement: S5 Table — For vector normalization, first and second differentiations were applied. PLS-DA, partial least squares discriminant analysis; Min-max, minimum-maximum; UV, unit variance; Par, pareto. (DOCX) [file pone.0186664.s011.docx]

**S5 Table. PLS-DA model parameters according to the number of components (one to three components), normalization (area, minimum-maximum, and vector normalization), and scaling methods (unit variance and pareto) for differentiation of ginseng parts using 6-year-old *Panax ginseng*.**

|  | **Normalization method** | **Scaling** | **R^2^Y** | **Q^2^Y** | **R^2^Y intercept** | **Q^2^Y intercept** |
| --- | --- | --- | --- | --- | --- | --- |
| **One component** | | | | | | |
| 6-year-old TR vs. RH vs. LR | Area | UV | 0.402 | 0.375 | 0.005 | -0.221 |
|  |  | Par | 0.362 | 0.330 | 0.002 | -0.198 |
|  | Min-max | UV | 0.384 | 0.354 | 0.003 | -0.214 |
|  |  | Par | 0.344 | 0.308 | 0.003 | -0.192 |
|  | Vector (first) | UV | 0.419 | 0.345 | 0.164 | -0.168 |
|  |  | Par | 0.373 | 0.317 | 0.081 | -0.157 |
|  | Vector (second) | UV | 0.488 | 0.355 | 0.129 | -0.245 |
|  |  | Par | 0.392 | 0.289 | 0.049 | -0.208 |
| **Two components** | | | | | | |
| 6-year-old TR vs. RH vs. LR | Area | UV | 0.723 | 0.554 | 0.134 | -0.308 |
|  |  | Par | 0.618 | 0.507 | 0.163 | -0.254 |
|  | Min-max | UV | 0.556 | 0.450 | 0.125 | -0.306 |
|  |  | Par | 0.595 | 0.520 | 0.163 | -0.248 |
|  | Vector (first) | UV | 0.889 | 0.800 | 0.362 | -0.340 |
|  |  | Par | 0.800 | 0.712 | 0.173 | -0.339 |
|  | Vector (second) | UV | 0.839 | 0.619 | 0.552 | -0.304 |
|  |  | Par | 0.678 | 0.501 | 0.265 | -0.317 |
| **Three components** | | | | | | |
| 6-year-old TR vs. RH vs. LR | Area | UV | 0.879 | 0.719 | 0.225 | -0.422 |
|  |  | Par | 0.889 | 0.758 | 0.256 | -0.370 |
|  | Min-max | UV | 0.823 | 0.682 | 0.264 | -0.340 |
|  |  | Par | 0.849 | 0.681 | 0.288 | -0.327 |
|  | Vector (first) | UV | 0.964 | 0.811 | 0.591 | -0.353 |
|  |  | Par | 0.922 | 0.810 | 0.405 | -0.394 |
|  | Vector (second) | UV | 0.944 | 0.782 | 0.786 | -0.220 |
|  |  | Par | 0.896 | 0.769 | 0.504 | -0.342 |

For vector normalization, first and second differentiations were applied. PLS-DA, partial least squares discriminant analysis; Min-max, minimum-maximum; UV, unit variance; Par, pareto.
