# Supplementary material for: Discrimination and prediction of cultivation age and parts of Panax ginseng by Fourier-transform infrared spectroscopy combined with multivariate statistical analysis
Source: PLoS One. 2017 Oct 19;12(10):e0186664. doi: 10.1371/journal.pone.0186664 (PMC5648215; doi:10.1371/journal.pone.0186664)
Supplement: S6 Table — Area normalization and two PLS components were used for discriminating between 5- and 6-year-old ginseng samples. TR, tap root; RH, rhizome; LR, lateral root; RMSEE, root mean squared error of estimation; RMSEP, root mean squared error of prediction; UV, unit variance; Par, pareto. (DOCX) [file pone.0186664.s012.docx]

**S6 Table.** **List of permutation parameters obtained by variables selected by various variable influence on projection (VIP) cutoff values and scaling methods.**

| **VIP cutoff** | **Total wavenumbers** | **RMSEE (months)** | **RMSEP (months)** | **R^2^Y** | **Q^2^Y** | **R^2^Y intercept** | **Q^2^Y intercept** | **Number of components** |
| --- | --- | --- | --- | --- | --- | --- | --- | --- |
| **5- vs. 6-year-old TR (UV scaling)** | | | | | | | | |
| 0 | 1478 | 0.185 (2.220) | 0.283 (3.396) | 0.904 | 0.719 | 0.345 | -0.362 | 2 |
| 1.0 | 537 | 0.177 (2.124) | 0.297 (3.564) | 0.913 | 0.825 | 0.262 | -0.271 | 2 |
| 1.3 | 322 | 0.165 (1.980) | 0.315 (3.780) | 0.923 | 0.856 | 0.162 | -0.289 | 2 |
| **5- vs. 6-year-old TR (Par scaling)** | | | | | | | | |
| 0 | 1478 | 0.173 (2.076) | 0.353 (4.236) | 0.916 | 0.649 | 0.335 | -0.345 | 2 |
| 1.0 | 538 | 0.151 (1.812) | 0.367 (4.404) | 0.936 | 0.754 | 0.245 | -0.378 | 2 |
| 1.3 | 194 | 0.257 (3.084) | 0.432 (5.184) | 0.815 | 0.756 | 0.194 | -0.354 | 2 |
| 1.5 | 125 | 0.220 (2.640) | 0.478 (5.736) | 0.865 | 0.790 | 0.193 | -0.345 | 2 |
| **5- vs. 6-year-old RH (UV scaling)** | | | | | | | | |
| 0 | 1478 | 0.207 (2.484) | 0.398 (4.776) | 0.880 | 0.816 | 0.390 | -0.276 | 2 |
| 1.0 | 567 | 0.199 (2.388) | 0.351 (4.212) | 0.889 | 0.835 | 0.352 | -0.282 | 2 |
| 1.3 | 32 | 0.163 (1.956) | 0.494 (5.928) | 0.926 | 0.774 | 0.293 | -0.240 | 2 |
| **5- vs. 6-year-old RH (Par scaling)** | | | | | | | | |
| 0 | 1478 | 0.224 (2.688) | 0.432 (5.184) | 0.860 | 0.794 | 0.340 | -0.278 | 2 |
| 1.0 | 426 | 0.212 (2.544) | 0.373 (4.476) | 0.874 | 0.788 | 0.290 | -0.182 | 2 |
| 1.3 | 296 | 0.277 (3.324) | 0.582 (6.984) | 0.786 | 0.694 | 0.081 | -0.237 | 2 |
| 1.5 | 220 | 0.301 (3.612) | 0.565 (6.780) | 0.747 | 0.644 | 0.074 | -0.258 | 2 |
| **5- vs. 6-year-old LR (UV scaling)** | | | | | | | | |
| 0 | 1478 | 0.146 (1.752) | 0.156 (1.872) | 0.940 | 0.774 | 0.416 | -0.287 | 2 |
| 1.0 | 407 | 0.158 (1.896) | 0.108 (1.296) | 0.930 | 0.836 | 0.283 | -0.362 | 2 |
| 1.3 | 262 | 0.171 (2.052) | 0.096 (1.152) | 0.918 | 0.806 | 0.231 | -0.296 | 2 |
| 1.5 | 69 | 0.219 (2.628) | 0.208 (2.496) | 0.865 | 0.750 | 0.162 | -0.291 | 2 |
| **5- vs. 6-year-old LR (Par scaling)** | | | | | | | | |
| 0 | 1478 | 0.166 (1.992) | 0.204 (2.448) | 0.923 | 0.798 | 0.378 | -0.296 | 2 |
| 1.0 | 518 | 0.263 (3.156) | 0.159 (1.908) | 0.806 | 0.562 | 0.225 | -0.245 | 2 |
| 1.3 | 209 | 0.358 (4.296) | 0.127 (1.524) | 0.641 | 0.498 | 0.078 | -0.250 | 2 |
| 1.5 | 138 | 0.313 (3.756) | 0.262 (3.144) | 0.726 | 0.598 | 0.209 | -0.226 | 2 |

Area normalization and two PLS components were used for discriminating between 5- and 6-year-old ginseng samples. TR, tap root; RH, rhizome; LR, lateral root; RMSEE, root mean squared error of estimation; RMSEP, root mean squared error of prediction; UV, unit variance; Par, pareto.
