# Supplementary material for: Discrimination and prediction of cultivation age and parts of Panax ginseng by Fourier-transform infrared spectroscopy combined with multivariate statistical analysis
Source: PLoS One. 2017 Oct 19;12(10):e0186664. doi: 10.1371/journal.pone.0186664 (PMC5648215; doi:10.1371/journal.pone.0186664)
Supplement: S7 Table — Minimum-maximum normalization and two PLS components were used for discriminating between 5- and 6-year-old ginseng samples. TR, tap root; RH, rhizome; LR, lateral root; RMSEE, root mean squared error of estimation; RMSEP, root mean squared error of prediction; UV, unit variance; Par, pareto. (DOCX) [file pone.0186664.s013.docx]

**S7 Table.** **List of permutation parameters obtained by variables selected by various variable influence on projection (VIP) cutoff values and scaling methods.**

| **VIP cutoff** | **Total wavenumbers** | **RMSEE (months)** | **RMSEP (months)** | **R^2^Y** | **Q^2^Y** | **R^2^Y intercept** | **Q^2^Y intercept** | **Number of components** |
| --- | --- | --- | --- | --- | --- | --- | --- | --- |
| **5- vs. 6-year-old TR (UV scaling)** | | | | | | | | |
| 0 | 1478 | 0.216 (2.592) | 0.223 (2.676) | 0.870 | 0.832 | 0.263 | -0.425 | 2 |
| 1.0 | 498 | 0.200 (2.400) | 0.396 (4.752) | 0.888 | 0.795 | 0.211 | -0.348 | 2 |
| 1.3 | 234 | 0.164 (1.968) | 0.563 (6.756) | 0.924 | 0.863 | 0.130 | -0.335 | 2 |
| 1.5 | 75 | 0.218 (2.616) | 0.090 (1.080) | 0.867 | 0.757 | 0.099 | -0.311 | 2 |
| **5- vs. 6-year-old TR (Par scaling)** | | | | | | | | |
| 0 | 1478 | 0.237 (2.844) | 0.188 (2.256) | 0.842 | 0.728 | 0.259 | -0.304 | 2 |
| 1.0 | 412 | 0.201 (2.412) | 0.086 (1.032) | 0.887 | 0.547 | 0.151 | -0.225 | 2 |
| 1.3 | 154 | 0.363 (4.356) | 0.436 (5.232) | 0.632 | 0.521 | 0.220 | -0.157 | 2 |
| 1.5 | 25 | 0.299 (3.588) | 0.512 (6.144) | 0.750 | 0.537 | 0.125 | -0.195 | 2 |
| **5- vs. 6-year-old RH (UV scaling)** | | | | | | | | |
| 0 | 1478 | 0.214 (2.568) | 0.095 (1.140) | 0.872 | 0.674 | 0.390 | -0.177 | 2 |
| 1.0 | 183 | 0.173 (2.076) | 0.114 (1.368) | 0.917 | 0.772 | 0.251 | -0.413 | 2 |
| 1.3 | 112 | 0.198 (2.376) | 0.036 (0.432) | 0.890 | 0.788 | 0.201 | -0.389 | 2 |
| 1.5 | 91 | 0.205 (2.460) | 0.076 (0.912) | 0.883 | 0.798 | 0.197 | -0.356 | 2 |
| 2.0 | 46 | 0.226 (2.712) | 0.426 (5.112) | 0.857 | 0.715 | 0.202 | -0.250 | 2 |
| **5- vs. 6-year-old RH (Par scaling)** | | | | | | | | |
| 0 | 1478 | 0.239 (2.868) | 0.196 (2.352) | 0.841 | 0.722 | 0.330 | -0.227 | 2 |
| 1.0 | 309 | 0.256 (3.072) | 0.209 (2.508) | 0.816 | 0.723 | 0.177 | -0.342 | 2 |
| 1.3 | 174 | 0.255 (3.060) | 0.242 (2.904) | 0.819 | 0.713 | 0.134 | -0.375 | 2 |
| 1.5 | 131 | 0.245 (2.940) | 0.255 (3.060) | 0.832 | 0.658 | 0.172 | -0.361 | 2 |
| 2.0 | 41 | 0.356 (4.272) | 0.218 (2.616) | 0.645 | 0.396 | 0.184 | -0.146 | 2 |
| **5- vs. 6-year-old LR (UV scaling)** | | | | | | | | |
| 0 | 1478 | 0.118 (1.416) | 0.279 (3.348) | 0.961 | 0.762 | 0.462 | -0.149 | 2 |
| 1.0 | 492 | 0.139 (1.668) | 0.343 (4.116) | 0.946 | 0.829 | 0.293 | -0.331 | 2 |
| 1.3 | 196 | 0.183 (2.196) | 0.411 (4.932) | 0.906 | 0.669 | 0.274 | -0.330 | 2 |
| 1.5 | 60 | 0.176 (2.112) | 0.307 (3.684) | 0.914 | 0.687 | 0.248 | -0.315 | 2 |
| **5- vs. 6-year-old LR (Par scaling)** | | | | | | | | |
| 0 | 1478 | 0.148 (1.776) | 0.332 (3.984) | 0.939 | 0.723 | 0.408 | -0.220 | 2 |
| 1.0 | 389 | 0.226 (2.712) | 0.437 (5.244) | 0.857 | 0.707 | 0.233 | -0.318 | 2 |
| 1.3 | 188 | 0.284 (3.408) | 0.520 (6.240) | 0.774 | 0.663 | 0.094 | -0.356 | 2 |
| 1.5 | 146 | 0.261 (3.132) | 0.479 (5.748) | 0.809 | 0.726 | 0.041 | -0.367 | 2 |
| 2.0 | 33 | 0.384 (4.608) | 0.697 (8.364) | 0.587 | 0.499 | 0.157 | -0.204 | 2 |

Minimum-maximum normalization and two PLS components were used for discriminating between 5- and 6-year-old ginseng samples. TR, tap root; RH, rhizome; LR, lateral root; RMSEE, root mean squared error of estimation; RMSEP, root mean squared error of prediction; UV, unit variance; Par, pareto.
