# Supplementary material for: Discrimination and prediction of cultivation age and parts of Panax ginseng by Fourier-transform infrared spectroscopy combined with multivariate statistical analysis
Source: PLoS One. 2017 Oct 19;12(10):e0186664. doi: 10.1371/journal.pone.0186664 (PMC5648215; doi:10.1371/journal.pone.0186664)
Supplement: S8 Table — Vector normalization after first differentiation and one PLS component were used for discriminating between 5- and 6-year-old ginseng samples. TR, tap root; RH, rhizome; LR, lateral root; RMSEE, root mean squared error of estimation; RMSEP, root mean squared error of prediction; UV, unit variance; Par, pareto. (DOCX) [file pone.0186664.s014.docx]

**S8 Table.** **List of permutation parameters obtained by variables selected by various variable influence on projection (VIP) cutoff values and scaling methods.**

| **VIP cutoff** | **Total wavenumbers** | **RMSEE (months)** | **RMSEP (months)** | **R^2^Y** | **Q^2^Y** | **R^2^Y intercept** | **Q^2^Y intercept** | **Number of components** |
| --- | --- | --- | --- | --- | --- | --- | --- | --- |
| **5- vs. 6-year-old TR (UV scaling)** | | | | | | | | |
| 0 | 1478 | 0.110 (1.320) | 0.131 (1.572) | 0.961 | 0.855 | 0.380 | -0.286 | 1 |
| 1.0 | 490 | 0.103 (1.236) | 0.075 (0.900) | 0.966 | 0.948 | 0.050 | -0.348 | 1 |
| 1.3 | 314 | 0.101 (1.212) | 0.088 (1.056) | 0.967 | 0.957 | -0.088 | -0.357 | 1 |
| 1.5 | 201 | 0.098 (1.176) | 0.089 (1.068) | 0.969 | 0.962 | -0.152 | -0.360 | 1 |
| **5- vs. 6-year-old TR (Par scaling)** | | | | | | | | |
| 0 | 1478 | 0.132 (1.584) | 0.156 (1.872) | 0.945 | 0.873 | 0.191 | -0.272 | 1 |
| 1.0 | 299 | 0.131 (1.572) | 0.081 (0.972) | 0.945 | 0.929 | -0.005 | -0.350 | 1 |
| 1.3 | 180 | 0.122 (1.464) | 0.095 (1.140) | 0.952 | 0.941 | -0.103 | -0.368 | 1 |
| 1.5 | 130 | 0.128 (1.536) | 0.097 (1.164) | 0.947 | 0.935 | -0.110 | -0.367 | 1 |
| 2.0 | 77 | 0.140 (1.680) | 0.115 (1.380) | 0.937 | 0.922 | -0.114 | -0.364 | 1 |
| 2.5 | 41 | 0.207 (2.484) | 0.108 (1.296) | 0.863 | 0.850 | -0.091 | -0.327 | 1 |
| **5- vs. 6-year-old RH (UV scaling)** | | | | | | | | |
| 0 | 1478 | 0.205 (2.460) | 0.227 (2.724) | 0.865 | 0.708 | 0.466 | -0.202 | 1 |
| 1.0 | 522 | 0.181 (2.172) | 0.203 (2.436) | 0.895 | 0.840 | 0.203 | -0.295 | 1 |
| 1.3 | 302 | 0.163 (1.956) | 0.200 (2.400) | 0.915 | 0.878 | 0.105 | -0.306 | 1 |
| 1.5 | 123 | 0.105 (1.260) | 0.157 (1.884) | 0.965 | 0.951 | -0.063 | -0.359 | 1 |
| **5- vs. 6-year-old RH (Par scaling)** | | | | | | | | |
| 0 | 1478 | 0.293 (3.516) | 0.324 (3.888) | 0.725 | 0.478 | 0.343 | -0.151 | 1 |
| 1.0 | 422 | 0.249 (2.988) | 0.283 (3.396) | 0.801 | 0.697 | 0.182 | -0.262 | 1 |
| 1.3 | 261 | 0.256 (3.072) | 0.270 (3.240) | 0.790 | 0.684 | 0.144 | -0.252 | 1 |
| 1.5 | 161 | 0.248 (2.976) | 0.226 (2.712) | 0.804 | 0.706 | 0.128 | -0.255 | 1 |
| 2.0 | 42 | 0.238 (2.856) | 0.084 (1.008) | 0.818 | 0.777 | 0.088 | -0.272 | 1 |
| **5- vs. 6-year-old LR (UV scaling)** | | | | | | | | |
| 0 | 1478 | 0.223 (2.676) | 0.664 (7.968) | 0.841 | 0.734 | 0.495 | -0.221 | 1 |
| 1.0 | 556 | 0.218 (2.616) | 0.678 (8.136) | 0.848 | 0.809 | 0.298 | -0.277 | 1 |
| 1.3 | 263 | 0.189 (2.268) | 0.633 (7.596) | 0.886 | 0.864 | 0.101 | -0.303 | 1 |
| 1.5 | 106 | 0.155 (1.860) | 0.602 (7.224) | 0.923 | 0.911 | -0.019 | -0.331 | 1 |
| **5- vs. 6-year-old LR (Par scaling)** | | | | | | | | |
| 0 | 1478 | 0.266 (3.192) | 0.653 (7.836) | 0.774 | 0.672 | 0.285 | -0.223 | 1 |
| 1.0 | 384 | 0.239 (2.868) | 0.690 (8.280) | 0.817 | 0.764 | 0.209 | -0.251 | 1 |
| 1.3 | 246 | 0.238 (2.856) | 0.672 (8.064) | 0.819 | 0.760 | 0.148 | -0.264 | 1 |
| 1.5 | 170 | 0.245 (2.940) | 0.679 (8.148) | 0.808 | 0.759 | 0.114 | -0.274 | 1 |
| 2.0 | 50 | 0.279 (3.348) | 0.656 (7.872) | 0.751 | 0.708 | 0.013 | -0.242 | 1 |

Vector normalization after first differentiation and one PLS component were used for discriminating between 5- and 6-year-old ginseng samples. TR, tap root; RH, rhizome; LR, lateral root; RMSEE, root mean squared error of estimation; RMSEP, root mean squared error of prediction; UV, unit variance; Par, pareto.
