# Supplementary material for: Discrimination and prediction of cultivation age and parts of Panax ginseng by Fourier-transform infrared spectroscopy combined with multivariate statistical analysis
Source: PLoS One. 2017 Oct 19;12(10):e0186664. doi: 10.1371/journal.pone.0186664 (PMC5648215; doi:10.1371/journal.pone.0186664)
Supplement: S9 Table — Vector normalization after second differentiation and one PLS component were used for discriminating between 5- and 6-year-old ginseng samples. TR, tap root; RH, rhizome; LR, lateral root; RMSEE, root mean squared error of estimation; RMSEP, root mean squared error of prediction; UV, unit variance; Par, pareto. (DOCX) [file pone.0186664.s015.docx]

**S9 Table.** **List of permutation parameters obtained by variables selected by various variable influence on projection (VIP) cutoff values and scaling methods.**

| **VIP cutoff** | **Total wavenumbers** | **RMSEE (months)** | **RMSEP (months)** | **R^2^Y** | **Q^2^Y** | **R^2^Y intercept** | **Q^2^Y intercept** | **Number of components** |
| --- | --- | --- | --- | --- | --- | --- | --- | --- |
| **5- vs. 6-year-old TR (UV scaling)** | | | | | | | | |
| 0 | 1478 | 0.065 (0.780) | 0.040 (0.480) | 0.986 | 0.918 | 0.412 | -0.309 | 1 |
| 1.0 | 552 | 0.077 (0.924) | 0.044 (0.528) | 0.981 | 0.970 | -0.064 | -0.369 | 1 |
| 1.3 | 331 | 0.085 (1.020) | 0.048 (0.576) | 0.977 | 0.967 | -0.154 | -0.356 | 1 |
| 1.5 | 131 | 0.095 (1.140) | 0.057 (0.684) | 0.971 | 0.961 | -0.201 | -0.387 | 1 |
| **5- vs. 6-year-old TR (Par scaling)** | | | | | | | | |
| 0 | 1478 | 0.092 (1.104) | 0.058 (0.696) | 0.973 | 0.907 | 0.123 | -0.321 | 1 |
| 1.0 | 268 | 0.100 (1.200) | 0.060 (0.720) | 0.968 | 0.956 | -0.156 | -0.364 | 1 |
| 1.3 | 187 | 0.112 (1.344) | 0.069 (0.828) | 0.960 | 0.945 | -0.169 | -0.365 | 1 |
| 1.5 | 150 | 0.118 (1.416) | 0.070 (0.840) | 0.956 | 0.939 | -0.176 | -0.370 | 1 |
| 2.0 | 87 | 0.114 (1.368) | 0.078 (0.936) | 0.959 | 0.942 | -0.175 | -0.364 | 1 |
| 2.5 | 44 | 0.118 (1.416) | 0.084 (1.008) | 0.955 | 0.937 | -0.199 | -0.382 | 1 |
| **5- vs. 6-year-old RH (UV scaling)** | | | | | | | | |
| 0 | 1478 | 0.093 (1.116) | 0.210 (2.520) | 0.972 | 0.792 | 0.412 | -0.228 | 1 |
| 1.0 | 523 | 0.075 (0.900) | 0.168 (2.016) | 0.982 | 0.948 | 0.193 | -0.342 | 1 |
| 1.3 | 278 | 0.059 (0.708) | 0.117 (1.404) | 0.989 | 0.976 | 0.031 | -0.340 | 1 |
| 1.5 | 136 | 0.051 (0.612) | 0.150 (1.800) | 0.992 | 0.986 | -0.081 | -0.353 | 1 |
| **5- vs. 6-year-old RH (Par scaling)** | | | | | | | | |
| 0 | 1478 | 0.188 (2.256) | 0.346 (4.152) | 0.887 | 0.586 | 0.204 | -0.162 | 1 |
| 1.0 | 387 | 0.136 (1.632) | 0.209 (2.508) | 0.941 | 0.860 | 0.107 | -0.306 | 1 |
| 1.3 | 236 | 0.198 (2.376) | 0.174 (2.088) | 0.874 | 0.771 | 0.087 | -0.271 | 1 |
| 1.5 | 158 | 0.239 (2.868) | 0.162 (1.944) | 0.816 | 0.703 | 0.056 | -0.257 | 1 |
| 2.0 | 61 | 0.336 (4.032) | 0.146 (1.752) | 0.639 | 0.555 | 0.002 | -0.215 | 1 |
| **5- vs. 6-year-old LR (UV scaling)** | | | | | | | | |
| 0 | 1478 | 0.252 (3.024) | 0.122 (1.464) | 0.796 | 0.558 | 0.727 | -0.128 | 1 |
| 1.0 | 499 | 0.249 (2.988) | 0.196 (2.352) | 0.801 | 0.709 | 0.354 | -0.255 | 1 |
| 1.3 | 283 | 0.241 (2.892) | 0.233 (2.796) | 0.815 | 0.742 | 0.165 | -0.268 | 1 |
| 1.5 | 162 | 0.225 (2.700) | 0.229 (2.748) | 0.837 | 0.787 | 0.043 | -0.278 | 1 |
| **5- vs. 6-year-old LR (Par scaling)** | | | | | | | | |
| 0 | 1478 | 0.318 (3.816) | 0.173 (2.076) | 0.677 | 0.417 | 0.515 | -0.115 | 1 |
| 1.0 | 381 | 0.292 (3.504) | 0.221 (2.652) | 0.726 | 0.604 | 0.222 | -0.238 | 1 |
| 1.3 | 232 | 0.324 (3.888) | 0.311 (3.732) | 0.664 | 0.552 | 0.109 | -0.218 | 1 |
| 1.5 | 167 | 0.329 (3.948) | 0.361 (4.332) | 0.653 | 0.557 | 0.080 | -0.215 | 1 |
| 2.0 | 71 | 0.360 (4.320) | 0.383 (4.596) | 0.586 | 0.508 | 0.040 | -0.178 | 1 |

Vector normalization after second differentiation and one PLS component were used for discriminating between 5- and 6-year-old ginseng samples. TR, tap root; RH, rhizome; LR, lateral root; RMSEE, root mean squared error of estimation; RMSEP, root mean squared error of prediction; UV, unit variance; Par, pareto.
