# Supplementary material for: Discrimination and prediction of cultivation age and parts of Panax ginseng by Fourier-transform infrared spectroscopy combined with multivariate statistical analysis
Source: PLoS One. 2017 Oct 19;12(10):e0186664. doi: 10.1371/journal.pone.0186664 (PMC5648215; doi:10.1371/journal.pone.0186664)
Supplement: S10 Table — Area normalization and three PLS components were used for discriminating ginseng samples from three parts (tap root, rhizome, lateral root). TR, tap root; RH, rhizome; LR, lateral root; RMSEE, root mean squared error of estimation; RMSEP, root mean squared error of prediction; UV, unit variance; Par, pareto. (DOCX) [file pone.0186664.s016.docx]

**S10 Table.** **List of permutation parameters obtained by variables selected by various variable influence on projection (VIP) cutoff values and scaling methods.**

| **VIP cutoff** | **Total wavenumbers** | **RMSEE (months)** | **RMSEP (months)** | **R^2^Y** | **Q^2^Y** | **R^2^Y intercept** | **Q^2^Y intercept** | **Number of components** |
| --- | --- | --- | --- | --- | --- | --- | --- | --- |
| **5-year-old TR vs. RH vs. LR (UV scaling)** | | | | | | | | |
| 0 | 1478 | 0.395 (4.740) | 0.057 (0.684) | 0.828 | 0.544 | 0.377 | -0.372 | 3 |
| 1.0 | 442 | 0.392 (4.704) | 0.033 (0.396) | 0.831 | 0.495 | 0.306 | -0.389 | 3 |
| 1.3 | 86 | 0.356 (4.272) | 0.104 (1.248) | 0.861 | 0.749 | 0.253 | -0.381 | 3 |
| 1.5 | 49 | 0.353 (4.236) | 0.145 (1.740) | 0.863 | 0.779 | 0.175 | -0.352 | 3 |
| **5-year-old TR vs. RH vs. LR (Par scaling)** | | | | | | | | |
| 0 | 1478 | 0.389 (4.668) | 0.127 (1.524) | 0.834 | 0.656 | 0.371 | -0.276 | 3 |
| 1.0 | 542 | 0.407 (4.884) | 0.059 (0.708) | 0.817 | 0.664 | 0.202 | -0.421 | 3 |
| 1.3 | 140 | 0.345 (4.140) | 0.171 (2.052) | 0.869 | 0.789 | 0.198 | -0.358 | 3 |
| 1.5 | 86 | 0.350 (4.200) | 0.174 (2.088) | 0.865 | 0.811 | 0.157 | -0.338 | 3 |
| **6-year-old TR vs. RH vs. LR (UV scaling)** | | | | | | | | |
| 0 | 1478 | 0.305 (3.660) | 0.092 (1.104) | 0.898 | 0.362 | 0.320 | -0.360 | 3 |
| 1.0 | 536 | 0.356 (4.272) | 0.177 (2.124) | 0.861 | 0.662 | 0.293 | -0.416 | 3 |
| 1.3 | 112 | 0.428 (5.136) | 0.240 (2.880) | 0.799 | 0.405 | 0.213 | -0.328 | 3 |
| **6-year-old TR vs. RH vs. LR (Par scaling)** | | | | | | | | |
| 0 | 1478 | 0.310 (3.720) | 0.138 (1.656) | 0.894 | 0.374 | 0.358 | -0.263 | 3 |
| 1.0 | 463 | 0.421 (5.052) | 0.373 (4.476) | 0.805 | 0.474 | 0.330 | -0.320 | 3 |
| 1.3 | 199 | 0.400 (4.800) | 0.314 (3.768) | 0.824 | 0.706 | 0.304 | -0.422 | 3 |
| 1.5 | 85 | 0.497 (5.964) | 0.277 (3.324) | 0.728 | 0.546 | 0.222 | -0.390 | 3 |

Area normalization and three PLS components were used for discriminating ginseng samples from three parts (tap root, rhizome, lateral root). TR, tap root; RH, rhizome; LR, lateral root; RMSEE, root mean squared error of estimation; RMSEP, root mean squared error of prediction; UV, unit variance; Par, pareto.
