# Supplementary material for: Discrimination and prediction of cultivation age and parts of Panax ginseng by Fourier-transform infrared spectroscopy combined with multivariate statistical analysis
Source: PLoS One. 2017 Oct 19;12(10):e0186664. doi: 10.1371/journal.pone.0186664 (PMC5648215; doi:10.1371/journal.pone.0186664)
Supplement: S11 Table — Minimum–maximum normalization and three PLS components were used for discriminating ginseng samples from three parts (tap root, rhizome, lateral root). TR, tap root; RH, rhizome; LR, lateral root; RMSEE, root mean squared error of estimation; RMSEP, root mean squared error of prediction; UV, unit variance; Par, pareto. (DOCX) [file pone.0186664.s017.docx]

**S11 Table.** **List of permutation parameters obtained by variables selected by various variable influence on projection (VIP) cutoff values and scaling methods.**

| **VIP cutoff** | **Total wavenumbers** | **RMSEE (months)** | **RMSEP (months)** | **R^2^Y** | **Q^2^Y** | **R^2^Y intercept** | **Q^2^Y intercept** | **Number of components** |
| --- | --- | --- | --- | --- | --- | --- | --- | --- |
| **5-year-old TR vs. RH vs. LR (UV scaling)** | | | | | | | | |
| 0 | 1478 | 0.378 (4.536) | 0.191 (2.292) | 0.843 | 0.444 | 0.412 | -0.210 | 3 |
| 1.0 | 333 | 0.401 (4.812) | 0.191 (2.292) | 0.823 | 0.450 | 0.380 | -0.360 | 3 |
| 1.3 | 125 | 0.448 (5.376) | 0.371 (4.452) | 0.779 | 0.317 | 0.364 | -0.316 | 3 |
| 1.5 | 85 | 0.565 (6.780) | 0.295 (3.540) | 0.649 | 0.110 | 0.351 | -0.221 | 3 |
| **5-year-old TR vs. RH vs. LR (Par scaling)** | | | | | | | | |
| 0 | 1478 | 0.315 (3.780) | 0.346 (4.152) | 0.891 | 0.485 | 0.403 | -0.240 | 3 |
| 1.0 | 422 | 0.380 (4.560) | 0.437 (5.244) | 0.841 | 0.532 | 0.318 | -0.283 | 3 |
| 1.3 | 193 | 0.363 (4.356) | 0.832 (9.984) | 0.855 | 0.745 | 0.273 | -0.263 | 3 |
| 1.5 | 128 | 0.377 (4.524) | 0.838 (10.056) | 0.844 | 0.738 | 0.272 | -0.258 | 3 |
| **6-year-old TR vs. RH vs. LR (UV scaling)** | | | | | | | | |
| 0 | 1478 | 0.350 (4.200) | 0.056 (0.672) | 0.865 | 0.567 | 0.387 | -0.288 | 3 |
| 1.0 | 530 | 0.435 (5.220) | 0.538 (6.456) | 0.792 | 0.299 | 0.351 | -0.230 | 3 |
| 1.3 | 107 | 0.594 (7.128) | 0.314 (3.768) | 0.612 | 0.330 | 0.246 | -0.281 | 3 |
| **6-year-old TR vs. RH vs. LR (Par scaling)** | | | | | | | | |
| 0 | 1478 | 0.398 (4.776) | 0.478 (5.736) | 0.826 | 0.240 | 0.414 | -0.210 | 3 |
| 1.0 | 329 | 0.446 (5.352) | 0.190 (2.280) | 0.782 | 0.431 | 0.273 | -0.312 | 3 |
| 1.3 | 206 | 0.443 (5.316) | 0.239 (2.868) | 0.784 | 0.536 | 0.235 | -0.342 | 3 |
| 1.5 | 101 | 0.460 (5.520) | 0.202 (2.424) | 0.767 | 0.367 | 0.221 | -0.327 | 3 |
| 2.0 | 29 | 0.922 (11.064) | 0.713 (8.556) | 0.065 | -0.277 | 0.255 | -0.106 | 3 |

Minimum–maximum normalization and three PLS components were used for discriminating ginseng samples from three parts (tap root, rhizome, lateral root). TR, tap root; RH, rhizome; LR, lateral root; RMSEE, root mean squared error of estimation; RMSEP, root mean squared error of prediction; UV, unit variance; Par, pareto.
