# Supplementary material for: Discrimination and prediction of cultivation age and parts of Panax ginseng by Fourier-transform infrared spectroscopy combined with multivariate statistical analysis
Source: PLoS One. 2017 Oct 19;12(10):e0186664. doi: 10.1371/journal.pone.0186664 (PMC5648215; doi:10.1371/journal.pone.0186664)
Supplement: S12 Table — Vector normalization after first differentiation and two PLS components were used for discriminating ginseng samples from three parts (tap root, rhizome, lateral root). TR, tap root; RH, rhizome; LR, lateral root; RMSEE, root mean squared error of estimation; RMSEP, root mean squared error of prediction; UV, unit variance; Par, pareto. (DOCX) [file pone.0186664.s018.docx]

**S12 Table.** **List of permutation parameters obtained by variables selected by various variable influence on projection (VIP) cutoff values and scaling methods.**

| **VIP cutoff** | **Total wavenumbers** | **RMSEE (months)** | **RMSEP (months)** | **R^2^Y** | **Q^2^Y** | **R^2^Y intercept** | **Q^2^Y intercept** | **Number of components** |
| --- | --- | --- | --- | --- | --- | --- | --- | --- |
| **5-year-old TR vs. RH vs. LR (UV scaling)** | | | | | | | | |
| 0 | 1478 | 0.247 (2.964) | 0.193 (2.316) | 0.927 | 0.788 | 0.626 | -0.293 | 2 |
| 1.0 | 604 | 0.269 (3.228) | 0.237 (2.844) | 0.913 | 0.819 | 0.507 | -0.308 | 2 |
| 1.3 | 132 | 0.198 (2.376) | 0.261 (3.132) | 0.953 | 0.916 | 0.410 | -0.266 | 2 |
| 1.5 | 23 | 0.204 (2.448) | 0.161 (1.932) | 0.950 | 0.913 | 0.352 | -0.223 | 2 |
| **5-year-old TR vs. RH vs. LR (Par scaling)** | | | | | | | | |
| 0 | 1478 | 0.440 (5.280) | 0.340 (4.080) | 0.767 | 0.588 | 0.376 | -0.247 | 2 |
| 1.0 | 391 | 0.282 (3.384) | 0.361 (4.332) | 0.904 | 0.624 | 0.295 | -0.297 | 2 |
| 1.3 | 241 | 0.331 (3.972) | 0.491 (5.892) | 0.869 | 0.611 | 0.253 | -0.302 | 2 |
| 1.5 | 174 | 0.401 (4.812) | 0.531 (6.372) | 0.807 | 0.677 | 0.162 | -0.287 | 2 |
| 2.0 | 47 | 0.611 (7.332) | 0.599 (7.188) | 0.553 | 0.415 | 0.200 | -0.196 | 2 |
| **6-year-old TR vs. RH vs. LR (UV scaling)** | | | | | | | | |
| 0 | 1478 | 0.151 (1.812) | 0.363 (4.356) | 0.972 | 0.917 | 0.667 | -0.238 | 2 |
| 1.0 | 550 | 0.181 (2.172) | 0.445 (5.340) | 0.960 | 0.931 | 0.534 | -0.292 | 2 |
| 1.3 | 175 | 0.202 (2.424) | 0.577 (6.924) | 0.951 | 0.894 | 0.432 | -0.238 | 2 |
| 1.5 | 66 | 0.204 (2.448) | 0.584 (7.008) | 0.946 | 0.892 | 0.342 | -0.183 | 2 |
| **6-year-old TR vs. RH vs. LR (Par scaling)** | | | | | | | | |
| 0 | 1478 | 0.225 (2.700) | 0.305 (3.660) | 0.939 | 0.881 | 0.422 | -0.255 | 2 |
| 1.0 | 408 | 0.218 (2.616) | 0.374 (4.488) | 0.943 | 0.913 | 0.359 | -0.337 | 2 |
| 1.3 | 236 | 0.259 (3.108) | 0.436 (5.232) | 0.920 | 0.886 | 0.329 | -0.366 | 2 |
| 1.5 | 149 | 0.301 (3.612) | 0.552 (6.624) | 0.891 | 0.859 | 0.267 | -0.352 | 2 |
| 2.0 | 48 | 0.401 (4.812) | 0.676 (8.112) | 0.807 | 0.726 | 0.190 | -0.320 | 2 |

Vector normalization after first differentiation and two PLS components were used for discriminating ginseng samples from three parts (tap root, rhizome, lateral root). TR, tap root; RH, rhizome; LR, lateral root; RMSEE, root mean squared error of estimation; RMSEP, root mean squared error of prediction; UV, unit variance; Par, pareto.
