# Supplementary material for: Discrimination and prediction of cultivation age and parts of Panax ginseng by Fourier-transform infrared spectroscopy combined with multivariate statistical analysis
Source: PLoS One. 2017 Oct 19;12(10):e0186664. doi: 10.1371/journal.pone.0186664 (PMC5648215; doi:10.1371/journal.pone.0186664)
Supplement: S13 Table — Vector normalization after second differentiation and two PLS components were used for discriminating ginseng samples from three parts (tap root, rhizome, lateral root). TR, tap root; RH, rhizome; LR, lateral root; RMSEE, root mean squared error of estimation; RMSEP, root mean squared error of prediction; UV, unit variance; Par, pareto. (DOCX) [file pone.0186664.s019.docx]

**S13 Table.** **List of permutation parameters obtained by variables selected by various variable influence on projection (VIP) cutoff values and scaling methods.**

| **VIP cutoff** | **Total wavenumbers** | **RMSEE (months)** | **RMSEP (months)** | **R^2^Y** | **Q^2^Y** | **R^2^Y intercept** | **Q^2^Y intercept** | **Number of components** |
| --- | --- | --- | --- | --- | --- | --- | --- | --- |
| **5-year-old TR vs. RH vs. LR (UV scaling)** | | | | | | | | |
| 0 | 1478 | 0.096 (1.152) | 0.185 (2.220) | 0.989 | 0.806 | 0.797 | -0.192 | 2 |
| 1.0 | 595 | 0.224 (2.688) | 0.137 (1.644) | 0.940 | 0.878 | 0.601 | -0.270 | 2 |
| 1.3 | 99 | 0.134 (1.608) | 0.187 (2.244) | 0.978 | 0.954 | 0.388 | -0.341 | 2 |
| **5-year-old TR vs. RH vs. LR (Par scaling)** | | | | | | | | |
| 0 | 1478 | 0.274 (3.288) | 0.465 (5.580) | 0.910 | 0.529 | 0.508 | -0.242 | 2 |
| 1.0 | 402 | 0.260 (3.120) | 0.597 (7.164) | 0.919 | 0.466 | 0.413 | -0.253 | 2 |
| 1.3 | 211 | 0.327 (3.924) | 0.759 (9.108) | 0.872 | 0.359 | 0.300 | -0.247 | 2 |
| 1.5 | 137 | 0.462 (5.544) | 0.601 (7.212) | 0.743 | 0.279 | 0.217 | -0.230 | 2 |
| 2.0 | 35 | 0.670 (8.040) | 0.557 (6.684) | 0.461 | 0.314 | 0.176 | -0.164 | 2 |
| **6-year-old TR vs. RH vs. LR (UV scaling)** | | | | | | | | |
| 0 | 1478 | 0.210 (2.520) | 0.106 (1.272) | 0.947 | 0.783 | 0.878 | 0.034 | 2 |
| 1.0 | 542 | 0.250 (3.000) | 0.215 (2.580) | 0.925 | 0.835 | 0.664 | -0.174 | 2 |
| 1.3 | 252 | 0.324 (3.888) | 0.298 (3.576) | 0.874 | 0.781 | 0.545 | -0.260 | 2 |
| 1.5 | 52 | 0.306 (3.672) | 0.252 (3.024) | 0.888 | 0.760 | 0.421 | -0.233 | 2 |
| **6-year-old TR vs. RH vs. LR (Par scaling)** | | | | | | | | |
| 0 | 1478 | 0.323 (3.876) | 0.128 (1.536) | 0.875 | 0.745 | 0.593 | -0.223 | 2 |
| 1.0 | 391 | 0.324 (3.888) | 0.206 (2.472) | 0.874 | 0.775 | 0.440 | -0.314 | 2 |
| 1.3 | 258 | 0.337 (4.044) | 0.185 (2.220) | 0.864 | 0.764 | 0.363 | -0.321 | 2 |
| 1.5 | 169 | 0.340 (4.080) | 0.229 (2.748) | 0.862 | 0.749 | 0.292 | -0.306 | 2 |

Vector normalization after second differentiation and two PLS components were used for discriminating ginseng samples from three parts (tap root, rhizome, lateral root). TR, tap root; RH, rhizome; LR, lateral root; RMSEE, root mean squared error of estimation; RMSEP, root mean squared error of prediction; UV, unit variance; Par, pareto.
